# Perceptions of HPV vaccination: a qualitative study with adolescents, parents, school staff, and pharmacists

**DOI:** 10.1186/s12916-026-04821-z

**Published:** 2026-03-26

**Authors:** Laurène Houtin, Lucia Romo, Yvonne Joret, Clémence Brun

**Affiliations:** 1 AD-HOC Lab, 23 Rue Hispano Suiza, Bois-Colombes, 92270 France; 2 2LPN (Psychology and Neuroscience Lab, UR7489), Université de Lorraine, Nancy, 54000 France; 3 Department of Psychology, University Paris Nanterre UR 4430, 200 Av de La République, Nanterre Cedex, 92001 France; 4 Institut pour le Développement et l’Action en Sciences Sociales (IDASC), 23 Rue Hispano Suiza, Bois-Colombes, France

**Keywords:** HPV vaccination, Health communication, Vaccine hesitancy, Adolescents, Parents, School staff, Pharmacists

## Abstract

**Background:**

Despite its proven efficacy in preventing HPV-related cancers, HPV vaccine uptake remains suboptimal in France (i.e., coverage reached 54.6% for girls and 15.8% for boys in 2023). Understanding stakeholder perspectives across key dimensions related to vaccination perceptions, HPV-related representations, barriers, facilitators, and communication dynamics is essential. This study investigates the knowledge, attitudes, barriers, and facilitators related to HPV vaccination among four stakeholder groups: adolescents, parents, school staff, and pharmacists.

**Methods:**

A total of 63 semi-structured interviews were conducted with middle school students (*n* = 15), parents (*n* = 18), school staff (*n* = 15), and pharmacists (*n* = 15) in France. Thematic analysis was used to identify common and group-specific representations, emotional responses, and social dynamics influencing HPV vaccine acceptance.

**Results:**

Participants generally supported HPV vaccination as a cancer-prevention tool but highlighted barriers such as limited HPV knowledge, gender-based misconceptions, unease discussing sexuality, concerns about side effects, and logistical hurdles. Facilitators included trust in healthcare professionals, pharmacies’ accessibility, and the school’s potential as an information platform. While adolescents expressed injection anxiety and a need for age-appropriate, anonymous formats, parents emphasized clear, data-driven communication. Overall, teachers felt underprepared, and pharmacists showed willingness to engage but required better informational tools. All groups called for targeted, emotionally resonant, multi-channel communication.

**Conclusions:**

Increasing HPV vaccination requires coordinated efforts that support schools, mobilize trusted healthcare professionals, and deliver clear, stigma-free information. Practical tools and targeted training could further strengthen stakeholder engagement.

**Supplementary Information:**

The online version contains supplementary material available at 10.1186/s12916-026-04821-z.

## HPV vaccination and public health impact

Human papillomavirus (HPV) is recognized as the primary cause of cervical cancer, which remains a significant global health concern. Cervical cancer is the fourth most common cancer in women worldwide and remains a major cause of preventable mortality, with the highest burden observed in low- and middle-income countries due to limited access to vaccination, screening, and treatment [1, 2]. In response, the World Health Organization launched an ambitious Cervical Cancer Elimination Initiative, with the goal of reducing cervical cancer incidence below 4 per 100,000 women in every country [1, 2].

A core pillar of this strategy is HPV vaccination, with a target of 90% of girls fully vaccinated by age 15 by the year 2030 [2]. Achieving this target, along with the complementary goals of screening 70% of women at least twice in a lifetime and ensuring that 90% of women with cervical disease receive appropriate treatment (i.e., the “90–70–90” targets; [3]), is projected to avert millions of cervical cancer cases and deaths over the next decades [1, 4]. Despite this global push, as of 2023, only about 20% of 15-year-old girls globally had received the full course of HPV vaccination [5]. This represents a modest improvement from previous years but highlights substantial disparities linked to variation in program delivery (e.g., school-based vs. clinic-based approaches), vaccine affordability and supply stability, competing health priorities, and differing levels of public confidence in vaccination [6–8]. Consequently, some countries achieve near-universal coverage (e.g., Cook Islands, Mexico, Seychelles), whereas others report substantially lower rates, remaining below global median estimates (e.g., Colombia, Armenia; [4]). The COVID-19 pandemic further disrupted routine immunization programs, causing declines in HPV vaccine delivery in many regions [5, 9–11]. Recent global estimates show that among girls aged 9–14 years, first-dose HPV vaccine coverage reaches 61.6%, whereas full two-dose coverage remains at 47.6%, based on pooled international data [12].

## HPV vaccination in Europe and in France: vaccination coverage and public health challenges

Europe has been a frontrunner in HPV vaccine introduction, yet coverage varies widely across the region. By 2023, the average HPV vaccination coverage in the European Union (EU) was about 56% of girls having received all recommended doses by age 15 [10]. Norway leads with 96% coverage among adolescent girls, and other Nordic countries like Sweden, Finland, and Denmark report similarly high rates (i.e., 80–90%) thanks to school-based vaccination and strong public health promotion [10, 13].

By contrast, several European countries struggle with low HPV vaccine uptake, and France has historically been among these lower-coverage countries [14]. HPV vaccination has been recommended and reimbursed at 65% by the French Health Insurance for adolescent girls since 2007, with the remaining cost generally covered by complementary private insurance [3, 14–16]. In 2019, the recommendation was expanded to include boys, and reimbursement at 65% for boys began in January 2021 [14, 16, 17]. The current guideline is for all adolescents irrespective of their gender aged 11–14 to receive a two-dose HPV vaccine series, with catch-up allowed up to 19 years. By 2018, only 24% of 16-year-old girls in France had completed the HPV vaccine series—a rate far below that of most high-income countries [14], and this figure represented a decline from earlier years (i.e., coverage had been about 30% in 2013 before falling amid controversy; [18]). This decline occurred in a context of public controversy: in late 2013, a highly publicized legal complaint was filed by a young woman who developed multiple sclerosis after HPV vaccination, followed by two additional complaints against the manufacturer. The extensive media coverage surrounding these cases fueled broader concerns about a potential link between HPV vaccination and autoimmune diseases, even though large-scale French surveillance data later confirmed the absence of increased risk [18, 19]. Recently, HPV vaccination coverage among adolescent girls in France has improved but remains below the European average. In 2023, 54.6% of 15-year-old girls had received at least one dose, and 44.7% of 16-year-olds were fully vaccinated [3], yet despite these gains, nearly half remain unprotected. As for boys, only 15.8% of 16-year-old boys were fully vaccinated against HPV in 2023 [20]. Coverage is especially low in southern and overseas regions (i.e., the territories located outside mainland Europe, such as Guadeloupe, Martinique, Réunion, and French Guiana), highlighting persistent health inequalities [3]. This shortfall has real consequences: worldwide, cervical cancer remains a major burden, with incidence and mortality reaching 13.3 and 7.3 per 100 000 women [21], and Europe reflects these global inequalities with pronounced regional contrasts, ranging from 7.0 and 2.0 per 100 000 in Western Europe to 14.5 and 6.1 per 100,000 in Eastern Europe [21]. France is no exception to this pattern, with about 3000 women diagnosed and 1100 dying each year despite the disease being largely preventable [3]. HPV also causes thousands of genital warts and precancerous lesions in both sexes, making improved vaccine uptake a key public health priority [3]. As such, the French National Authority for Health (Haute Autorité de Santé, HAS) is considering extending catch-up HPV vaccination for females and males up to 26 years of age, to further extend vaccine coverage [22].

## Barriers to HPV vaccination in France and stakeholders perspectives

Until 2023, the absence of a school-based program created a cumbersome multi-step process for families involving doctor visits, pharmacy pickups, and injections, contrasting with more streamlined approaches in countries like the UK and Portugal [15, 23]. This complexity was exacerbated by widespread vaccine hesitancy, fueled by media controversies surrounding alleged side effects and a general climate of public doubt, with 41% of the population expressing mistrust in vaccines by 2016 [24–26]. Although health authorities responded with campaigns and the inclusion of HPV vaccination in the national Cancer Plan (2014–2019), coverage targets were not met [23]. Recent changes, however, mark a turning point. In 2023, France launched its first school-based HPV campaign for 12-year-olds in seventh grade, delivered by mobile teams with parental consent [20]. Initial results are promising: one-dose coverage at age 15 doubled between 2022 and 2023 (from approximately 13% to 26%) [27], and the program is set to continue. Access has also been expanded through pharmacists and nurses, alongside broader communication efforts [27]. Understanding the views of key French stakeholders—adolescents, parents, school staff, and healthcare professionals—is critical to guide effective interventions.

Parental knowledge and attitudes are crucial determinants of adolescent vaccination, yet many French parents are poorly informed, with up to one-third having never heard of HPV and only a minority understanding the vaccine’s preventive role [28]. Safety concerns are a major driver of hesitancy, with most refusers fearing side effects despite strong evidence of vaccine safety [25, 28]. Some parents perceive HPV risk as distant, either because they believe their child is too young or because cervical cancer feels like a far-off concern. Others worry that receiving the HPV vaccine might encourage early sexual activity [23, 28, 29]. Socioeconomic disparities further complicate the issue, with higher uptake observed among wealthier, more educated families [13, 28]. These disparities are partly explained by differences in access to vaccination services: areas with higher socioeconomic status tend to offer greater availability of healthcare resources, which is associated with higher uptake [30]. Recent data from China also indicate that HPV-related knowledge and vaccination are more common among families with higher socioeconomic status, partly because these families are better able to mobilize social and financial resources to access vaccination, while the cost remains prohibitive for many low-income households [31]. In this context of socioeconomic differences, the influence of healthcare providers is even more critical, as many parents base their decisions on their recommendations [25, 32, 33]. While French GP (general practitioner) and pediatricians are central to vaccine uptake, their engagement has been inconsistent [24]. In 2014, over 25% of healthcare professionals believed that some recommended vaccines, including the HPV vaccine, were not useful [34]. This negative perception of the HPV vaccine was often due to safety doubts, time constraints during consultations, communication challenges, or dose scheduling issues [29, 35–37]. Although the COVID-19 pandemic appears to have fostered a more favorable view of the HPV vaccine—especially for boys—it does not seem to have altered how these barriers are perceived [38]. Strong, repeated recommendations and addressing parental concerns, including personal endorsements, are key strategies to enhance credibility [23, 39]. Previous studies have shown that pharmacists can play a key role in promoting and administering vaccination, given their accessibility, existing infrastructure, and recognized skills in vaccine administration [40–43]. However, to effectively support their commitment, adequate resources are essential: training, computer alerts, supportive materials, and the implementation of vaccination services within pharmacies [42]. As schools become more actively involved in the vaccination process [44], school personnel (e.g., teachers, nurses, principals) are increasingly engaged. While 90% of school staff know about the HPV vaccine, many lack detailed knowledge of its broader consequences (e.g., genital warts, oropharyngeal cancers; [14, 45]), mirroring teenagers’ reports of inadequate or unclear information about HPV vaccination [25]. Safety concerns and discomfort discussing sexually transmitted infection (STI)-related topics with students are common among school staff [14, 32]. Some teachers also fear parental backlash, feel undertrained, or consider health communication outside their role [45]. In contrast, school nurses are generally more knowledgeable and view HPV education as part of their responsibilities, yet they often lack sufficient training and resources to address it effectively [14, 32]. Thus, these persistent knowledge and confidence gaps highlight the limits of logistical progress alone. Understanding the perspectives of key French stakeholders—adolescents, parents, school staff, and healthcare professionals—is essential to guide effective future interventions.

## Background

This qualitative study is unique because it explores HPV vaccine acceptance by examining and cross-referencing the perceptions, barriers, and drivers identified across four key stakeholder groups—middle school students, parents, school staff (i.e., teachers) and pharmacists. By bringing together these typically separate perspectives on vaccine representations, this research aims to identify particularly relevant and coherent intervention strategies grounded in the realities of the school environment.

## Methods

### Study design and data collection

A qualitative design was chosen to gain in-depth insights into the representations, emotional reactions, and social influences surrounding HPV vaccination, particularly in the context of recent school-based campaigns in France. Semi-structured interviews were selected because they allow researchers to explore participants’ perspectives in depth while ensuring that key topics are systematically addressed [46].

We followed the five-step framework proposed by Kallio et al. [47] to develop our semi-structured interview guides. According to this framework, researchers should (i) ensure that semi-structured interviewing is appropriate for exploring the phenomenon of interest. This was the case in our study, which required accessing participants’ perceptions, emotions, and decision-making processes regarding HPV vaccination. Kallio et al. [47] also recommend (ii) grounding the guide in existing knowledge. We therefore drew on the literature on HPV vaccination, vaccine hesitancy, and adolescent health to identify the main thematic areas. They further advise (iii) drafting a preliminary guide composed of open, non-leading questions organized from general to more sensitive topics. We followed this recommendation by developing group-specific guides structured from broad questions about vaccination to more specific issues related to HPV, sexuality, and the school-based campaign. The framework also highlights the importance of (iv) testing and refining the guide before data collection. In line with this, the guides were reviewed within the research team and then read by two individuals from each target population in our personal networks, who provided external feedback that informed adjustments to wording and question order. Finally, Kallio et al. [47] stress (v) ensuring transparency by reporting the final guide, as such the complete interview guides are included in the Supplementary Material.

The student interview guide explored:

- Knowledge and understanding of vaccination, including past vaccination experiences,
- Awareness and opinions regarding the HPV vaccine, and associated feelings (e.g., unfamiliarity, fear, confidence),
- Sources of information and influences on vaccination decisions, including the role of adults (i.e., parents, teachers, healthcare providers) and peers,
- Reasons for accepting or refusing vaccination,
- Perceptions of the role of schools and healthcare professionals in vaccination efforts.

The parent interview guide focused on:

- General knowledge and attitudes toward vaccination, and specific awareness and reactions to the HPV vaccine,
- Decision-making factors influencing HPV vaccination choices, including any doubts or concerns,
- The influence of healthcare provider recommendations and social interactions with other parents,
- Perceptions of the school’s role in providing information and organizing vaccination campaigns,
- Information needs and preferences for communicating with families about HPV vaccination.

The school staff interview guide investigated:

- Perceptions of the HPV vaccine, and related information or training received,
- Views on the school’s role in HPV vaccination information and promotion, and any prior involvement in these efforts,
- Observed attitudes and reactions among students and families, and the prevalence of positive or negative discourse,
- Needs for additional resources, training, or support, and suggestions for improving information dissemination and building trust in the vaccine.

The pharmacist interview guide examined:

- Professional roles and perceived evolution in HPV vaccination, including observed changes in client demand and attitudes,
- Interactions with clients regarding HPV vaccine-related questions and concerns, and strategies for addressing hesitancy,
- Perceived adequacy of training and support for addressing client inquiries and promoting vaccination,
- Perceived obstacles and facilitators in daily practice, and effective strategies for reassuring or persuading hesitant individuals.

Interviews were conducted between April and June 2025 by trained members of the research team. All interviews were conducted remotely via Google Meet [48], in line with participant preferences, each session being scheduled to last between 20 and 45 min. With participants’ consent, all interviews were audio-recorded and transcribed verbatim.

### Participants and recruitment

The study included adolescents, parents, school staff, and pharmacists. Adolescents and parents were recruited through the Bilendi [49] paneling platform^1^ (i.e., €50 compensation per interview) and snowball sampling, while some school staff (i.e., teachers from various disciplines) and pharmacists were recruited via LinkedIn messages [50], followed by personalized invitations to participate in the study in exchange for a €50 gift card.^2^ Inclusion criteria were as follows: (a) being a parent of a child aged 11–14 enrolled in a participating school; (b) being a student aged 11–14 enrolled in a participating school; (c) being a teacher or healthcare staff working in a middle school; (d) being a community pharmacist practicing in France. All participant groups (i.e., adolescents, parents, teachers, pharmacists) were recruited independently, with no dyadic or institutional linkage between respondents.

The study adhered to ethical guidelines for social science research, ensuring participant anonymity and the right to withdraw at any time and adhering to national and institutional guidelines, including the Helsinki Declaration. All participants provided written informed consent, with parental consent obtained for minors. The consent forms are available as Supplementary Material.

### Data analysis

We analyzed the interview data using template analysis [51, 52], a form of thematic analysis that emphasizes the development of a hierarchical coding template and its iterative refinement. Consistent with qualitative approaches that seek to understand how participants make sense of their experiences [53, 54], the analysis was conducted within an interpretivist qualitative paradigm and included reflexive considerations regarding the researchers’ assumptions, documented through brief analytic memos and regular debriefing discussions. Central to this approach is the construction of an initial template on the basis of a subset of the data, which is then applied to further material and revised as necessary. In our study, the initial coding combined themes that had been identified a priori when designing the interview guides with additional codes that were generated from the transcripts, in accordance with the flexibility offered by template analysis, which explicitly permits, but does not require, the use of a priori themes. After repeated reading of the interviews, two trained qualitative researchers independently coded all transcripts, highlighting all segments relevant to our research questions and assigned preliminary codes before meeting to compare and discuss their interpretations line by line until reaching a negotiated consensus, as recommended for interpretivist qualitative research rather than relying on inter-rater reliability metrics [55]. These codes were then grouped into more abstract categories and organized into a hierarchical structure, with narrower subthemes nested within broader themes. Coding continued until no substantively new themes emerged. A subset of interviews from each participant group was used as a pilot phase to refine the preliminary coding template before applying it to the full dataset [52], allowing for the clarification of code boundaries, the addition of emergent subthemes, and the reorganization of the template’s structure where needed. Throughout this process, we drew on Braun and Clarke’s [54] guidelines for thematic analysis, moving from familiarization with the data, through coding and theme development, to reviewing and refining the thematic structure, to ensure that our analysis remained systematic and transparent. Data organization and coding were supported by NVivo 15 [56], which was also used to store analytic memos and facilitate iterative revisions to the coding template. Anonymized verbatim extracts were selected to illustrate each major theme and to reflect the range of perspectives observed across participant groups. These quotes were chosen based on their representativeness of observed opinion trends and the level of information they offered regarding our objectives. The final thematic structure is summarized in Table 1.

**Table 1:** Summary of main themes and sub-themes by group (parents, adolescents, teachers, pharmacists)

Group	Main themes	Sub-themes
Parents	Vaccination as a normative yet unevenly embodied public health practice	Trust in recommendations, concerns about side effects (M)^a^
	HPV vaccination caught between fragmented knowledge, gendered representations, and late awareness	Late discovery of the vaccine, gender stereotypes (B)
	When prevention collides with fear, stigma, and institutional uncertainty	Age perceived as too young, fear of side effects, misinformation (B)
		Few peer discussions, topic perceived as taboo (B)
	Trust, proximity, and legitimacy as key levers for vaccine acceptance	Cancer prevention, personal experience (F)
		Importance of medical recommendations, lack of initiative from physicians (M)
	From information gaps to demands for credible, emotionally attuned communication	School campaigns seen as useful but uneven, role of communication (M)
		Lack of accessible information, expectations toward institutions and influencers (M)
Adolescents	Vaccination as a normative yet unevenly embodied public health practice	Understanding of prevention, fear of needles among some (M)
	HPV vaccination caught between fragmented knowledge, gendered representations, and late awareness	Frequent lack of knowledge, gender stereotypes, uncertainty about transmission (B)
	When prevention collides with fear, stigma, and institutional uncertainty	Pain, side effects, discomfort discussing sexuality, parental opposition (B)
	Trust, proximity, and legitimacy as key levers for vaccine acceptance	Cancer prevention, advice from relatives, sense of shared responsibility (F)
	From information gaps to demands for credible, emotionally attuned communication	Family and doctor as primary sources, limited peer discussion (M)
		Sessions seen as insufficient or awkward, strong demand for improvement (M)
		Small group sessions, anonymous questions, engaging formats (F)
Teachers	Vaccination as a normative yet unevenly embodied public health practice	Widespread support, some doubts due to lack of information (M)
	HPV vaccination caught between fragmented knowledge, gendered representations, and late awareness	Fragmented knowledge, confusion over vaccination of boys (B)
	When prevention collides with fear, stigma, and institutional uncertainty	Lack of training, discomfort around sexuality, fear of parental reactions (B)
	Trust, proximity, and legitimacy as key levers for vaccine acceptance	Informing rather than vaccinating, need for coordination with healthcare (M)
		Medical interventions, official educational materials, stronger coordination (F)
	From information gaps to demands for credible, emotionally attuned communication	Dedicated training, concrete tools, institutional communication (M)
Pharmacists	Vaccination as a normative yet unevenly embodied public health practice	Recent increase in vaccination, impact of public campaigns (F)
	HPV vaccination caught between fragmented knowledge, gendered representations, and late awareness	Confusion about boys’ vaccination (B)
	When prevention collides with fear, stigma, and institutional uncertainty	Side effects, injection pain (B)
		HPV trivialization, post-COVID vaccine hesitancy, logistical challenges (B)
	Trust, proximity, and legitimacy as key levers for vaccine acceptance	Recognized central role, complementarity with physicians (F)
		Accessibility in pharmacies, clear messaging, trust (F)
	From information gaps to demands for credible, emotionally attuned communication	Neutral communication, risk–benefit framing, trust-based relationship (F)
		Regular training, printed tools, sample counseling scripts (M)

^a^Role codes: *(F*) facilitator, *(B)* barrier, *(M)* mixed

## Results

The final sample comprised 15 adolescents (*M*_Age_ = 13.00 years, *SD*_Age_ = 0.98; 46.67% girls), 18 parents (*M*_Age_ = 47.78 years, *SD*_Age_ = 8.98; 66.67% mothers), 15 school staff (*M*_Age_ = 40.07 years, *SD*_Age_ = 9.52, 80.00% women, 13 specified their subject: 3 Spanish teachers, 1 English teacher, 1 history teacher, 2 biology teachers, 3 mathematics teachers, 1 French/FLS teacher, and 2 physical education teachers), and 15 pharmacists (*M*_Age_ = 41.27 years, *SD*_Age_ = 9.74, 46.67% women).

### Vaccination as a normative yet unevenly embodied public health practice

The majority of parents interviewed (16 out of 18) expressed a generally positive view of vaccination. They regarded it as important for preventing disease and protecting their children, and reported trusting public health recommendations: *“When the doctor and the health authorities tell us it’s something we should do because it’s necessary, I have to admit I don’t really question it—I just follow the recommendations”* (Interview 2). Only a minority of parents (2 out of 18) expressed stronger reservations about vaccines in general, citing for example the sense that “there are too many vaccines” and concerns about potential side effects: *“I felt she was a bit young for that kind of vaccine. I mean, they’re already given so many vaccines… Yeah, I’m not really in favor of it”* (Interview 15).

Compared with parents, adolescents expressed confidence for similar reasons (i.e., trust in adults and prevention), but their doubts were more strongly shaped by personal experiences and peer-circulated rumors. All of the middle school students interviewed were able to articulate, in broad terms, what a vaccine is, its function, and its purpose, which they defined as a method of disease prevention that “trains” the body to defend itself. Several participants used their own language to describe vaccination as “*something you do with a shot… so you don’t get sick*” (Interview 39), or as “*introducing tiny doses of germs*” (Interview 50). One student remarked that “*it gives me the disease but in a very mild form so that I get used to it and can fight the virus more easily*” (Interview 36). This preventive dimension was well grasped by the majority of respondents. However, a minority of pupils expressed general mistrust toward vaccines. Three out of fifteen adopted a consistently skeptical stance, often grounded in negative personal experiences or alarming information. For instance, one 13-year-old male student recounted having “*had a vaccine once and then getting sick right afterwards*,” which led him to say, “*honestly, now I don’t really trust*” vaccines (Interview 44). This pupil further echoed online rumors—“*we don’t even know what’s in them. Maybe it’s aluminum, or stuff to monitor people. Some guy on TikTok even said it could change your DNA*”—rumors that are scientifically unfounded but circulate widely on social media. He also expressed distrust toward pharmaceutical companies (“*The labs just want to make money*”; Interview 44).

Teachers likewise expressed high confidence in vaccines, but unlike parents and adolescents, they grounded their views mainly in collective health benefits rather than personal experience. A large majority of teachers hold a very favorable view of vaccines overall. Indeed, 13 out of 15 teachers identify as broadly pro-vaccination, acknowledging the collective health benefits conferred by immunization. Several respondents emphasized the historical impact of vaccines in eradicating once‐prevalent diseases. As one participant noted, “*most people come to understand that measles, rubella, and similar diseases… have been successfully eradicated thanks to vaccination*” (Interview 4). This predominant confidence in vaccination is accompanied by a sense of civic and educational responsibility to engage in preventive measures. Nevertheless, 2 out of 15 teachers adopt a more conflicted stance: “*I don’t see the risk. So getting vaccinated for something… if you don’t have the disease or the problem, I don’t really see the point. I don’t perceive how serious it is—or isn’t—and what the actual likelihood is of getting it*” noted a 26-year-old female teacher (Interview 49). They express lower confidence, stemming from a perceived lack of clear information or the coexistence of contradictory messages. As one teacher summarized, “*Well, I think we lack information overall, because you hear both good and bad things*” (Interview 9).

Pharmacists also expressed strong support for vaccination, though their perspective was shaped by their hands-on involvement and their proximity to patients. All pharmacists interviewed expressed a positive perception of their role in HPV vaccination. They see themselves as key players due to their proximity to patients, their ability to provide accurate and reassuring advice, their capacity to directly administer the vaccine, and the growing recognition of their role through evolving legislation and increasing numbers of prescriptions: “*We really have a laboratory that’s trying to implement this vaccination, along with the authorities who are supporting it […] So of course, we’re getting more prescriptions, and of course, we’re being approached more often about it. So our role is central—and that’s exactly how it should be*” (Interview 52), “*I think that in the future, especially in a strained healthcare context, doctors will focus more on their core medical role, and for procedures that—while medical in nature—we are trained to perform, we’ll be able to take over*” (Interview 60). As one participant emphasized, “*We have an essential role in advising on this vaccine […] We are in frequent contact with mothers, adolescents… young girls, and young boys as well*” (Interview 58). In this sense, pharmacists have observed that their patients clearly understand their dual role as prescribers and vaccinators, and they see themselves as well-positioned to reach patients whose physicians may not have informed them about the HPV vaccine: “*Then, when we’re dealing with doctors who don’t necessarily think about it, it’s true that it’s up to us to initiate the conversation and bring it up. You need to have the reflex, when you get a prescription at the counter, to check the patient’s age and talk to the parents […] So it’s partly our job to catch what might have slipped through the cracks and been forgotten*” (Interview 55). If they are concerned about not being seen as credible, they draw on their complementary role alongside physicians to encourage patients to reflect and, ultimately, to support vaccination: “*And whenever someone seems unconvinced or skeptical, I tell them: “The pharmacist mentioned this vaccine—talk to your doctor about it. They’ll either give you a prescription or say, ‘Yes, go ahead and get vaccinated at the pharmacy, that’s perfectly fine’”*” (Interview 64). As such, these distinct ways of justifying vaccination appear to orient how each group engages with HPV prevention: adolescents’ reasoning remains largely situational and experience-based, whereas parents and professionals refer to more formalized frameworks of responsibility.

### HPV vaccination caught between fragmented knowledge, gendered representations, and late awareness

Fourteen out of 18 parents had at least a vague awareness of the HPV before vaccination—usually through the internet, media, relatives, or healthcare professionals—without necessarily understanding the full details: *“I’ve heard about it for a while. I know there are campaigns. I’ve seen them on television, I think I’ve seen them online too. I even saw some information in doctors’ offices, in waiting rooms. So yes, I’ve heard about it”* (Interview 3). However, 4 out of 18 parents discovered the existence of the vaccine only very recently, for example, during a school-based vaccination campaign: *“Oh yeah, otherwise I hadn’t heard of it at all. And it’s thanks to the school—they gave us documents to read so we could decide whether or not to do it”* (Interview 2). The fact that several parents only became aware of the HPV vaccine through school-based initiatives suggests that HPV prevention is not yet embedded in routine parental health monitoring, but rather encountered as an external prompt, often at a late stage. This late discovery was sometimes accompanied by surprise regarding the nature of the virus. Several parents initially believed that HPV was transmitted only through sexual contact and concerned only girls. One parent noted: *“We thought it only affected girls, generally speaking, but in fact, from the various studies we saw and what was explained to me, it affects boys too”* (Interview 1). This belief reflects a persistent gendered framing of HPV as a “female” issue, closely tied to representations of sexuality and reproductive health, which contributes to the marginalization of boys in vaccination discourse. This process of learning led some to realize that HPV is a public health issue that can potentially affect anyone: *“I didn’t know there were types of HPV you could catch directly. I thought it was only an STD. But no—it can be caught through other types of infection, like from warts, or even at the swimming pool. Like at the municipal pool,*^3^* for instance”* (Interview 1). Overall, the idea of vaccinating against HPV was positively received. A large majority of parents (15 out of 18) welcomed the opportunity to have their child vaccinated, viewing it as an important step in preventing serious diseases, particularly certain cancers. For instance, a father who had experienced cancer in his family described it as a decisive factor: *“My wife has cancer […] so that had a direct impact on our decision. And seeing that HPV could lead to cancer later in life, for both girls and boys, we wanted to reduce that risk for our children—otherwise, if they got cancer and we’d done nothing, we’d regret it as parents”* (Interview 1). More broadly, 14 out of 18 parents felt it was better to act now while a preventive option was available, in line with their responsibility to protect their children: *“If I can protect her, I will”* (Interview 11). For some, the decision felt almost instinctive, particularly if they or people close to them had already been vaccinated: *“My gynecologist told me about it a few years ago, so I got vaccinated myself. Then it just felt natural for my daughter to get it. It’s safe, no side effects, and it’s protective—it’s all positive”* (Interview 3). Beyond these intrinsic motivations, one mother mentioned the role of official communication in prompting her to consider the vaccine: *“I think it was the CPAM*^4^* that sent me an email about preventing HPV. That got my attention. And since the email was aimed at my daughter’s age group, I thought, ‘Maybe it’s something we should discuss as a family’”* (Interview 20). In this context, access to clear information served as a real motivator: *“What convinced me was the percentage showing you can’t get the disease if you’re vaccinated”* (Interview 2). Some parents also stressed the importance of explaining vaccination to children themselves: *“The reason behind the vaccine—that’s really what matters most: why are we getting vaccinated? So that I can explain it to my kids too”* (Interview 7). Only 2 parents expressed a more cautious initial reaction, in line with their general reluctance toward vaccination, approaching the HPV vaccine with more questions and hesitation. Recommendations from healthcare professionals were a decisive factor for many parents. In 12 out of 18 interviews, parents stated that they chose to have their child vaccinated (or decided to do so) primarily based on the advice of their physician, in whom they placed strong trust: *“So I trust my general practitioner—he explained it to me directly without pushing me. He told me, ‘It’s your choice, but as a doctor I advise you to do it.’ So I followed his advice”* (Interview 1). However, several parents expressed regret that healthcare professionals did not take more initiative in raising the topic of HPV vaccination. In 6 out of 18 interviews, parents noted that neither their family doctor nor their child’s pediatrician had mentioned the HPV vaccine during routine check-ups. In some cases, it was up to the parents to raise the subject themselves in order to get information or a prescription, once they had learned about the vaccine through other channels. One father recounted that his doctor had never brought it up until he specifically asked about his son: *“I think that if I hadn’t asked, ‘What about boys?’, I might never have vaccinated my eldest”* (Interview 1). More generally, many parents expressed the desire for a more proactive role from the medical community in promoting the HPV vaccine. Twelve parents indicated that they would have appreciated it if the topic had been addressed more systematically by their doctor during routine visits. They believed that primary care physicians should incorporate HPV prevention into the regular medical follow-up of children at key ages, so as not to miss any opportunity to inform families: *“Yeah, I think the doctor has a role to play too. If doctors were made more aware, then when they see a teenager, they could just ask: ‘Have you been vaccinated yet? It might be a good idea—it’s effective protection’”* (Interview 20). Others mentioned the possibility of involving pharmacists—both for advice and for administering the vaccine: *“Or we could just go see the pharmacist. I think some pharmacists are allowed to give the vaccine, like they did for COVID”* (Interview 17). In contrast, the influence of relatives or friends appeared to be a much less decisive factor. Only 3 out of 18 parents reported having discussed the HPV vaccine with other parents within their family or social circle. In these rare cases, conversations with peers sometimes helped confirm their decision—especially when they learned that others had had their children vaccinated without issue—but could also expose them to more hesitant views. One participant noted, for example, that some members of her family had expressed reluctance to vaccinate a boy, although this did not prevent her from vaccinating her own children. Overall, very few parents cited the opinion of people around them as something that had significantly influenced their decision—particularly when it came to those outside the family circle. For many, HPV and sexual health remained delicate or taboo subjects: *“To be honest, it’s like talking about condoms—you’re not really going to bring it up with your friends. These are topics that feel a bit taboo, about diseases we might catch… They’re things we don’t even talk about within the family. It really stays in a very close circle”* (Interview 1). This quote illustrates how sexuality-related taboos restrict informal discussion and information circulation, thereby reinforcing silence and limiting opportunities for normalization of HPV vaccination.

Compared with parents, adolescents showed even wider variation in awareness, ranging from basic familiarity to complete discovery during the interview. The extent of adolescents’ awareness of HPV and its vaccine varied considerably. Two-thirds of the students (10/15) reported having heard of HPV prior to the interview. Among those who were informed, many remained vague about its nature: some understood it simply as “*a virus that can cause cancers*” (Interview 43), typically discussed in biology class under the topic of sexually transmitted infections. One 9th-grade student even noted that “*you can catch it without necessarily realizing it*,” which she found “*a bit unsettling*” (Interview 43). Others associated the term only with “*a virus… kind of related to sex*” without further detail (Interview 45). In contrast, one-third of the students (5/15) had never heard of HPV before. When introduced to the idea of an HPV vaccine, these pupils often expressed surprise and mistrust toward this “unknown”: “*I heard that name once, but I don’t know what it is*,” admitted one student (Interview 36), while another wondered, “*Is it even a real disease?*” (Interview 39). These gaps in knowledge are partly explained by limited exposure to the topic outside of recent public health campaigns. Moreover, certain misconceptions persisted, notably the belief that the HPV vaccine is intended only for girls. One mother allegedly told her son “*it wasn’t for boys*” (Interview 42), discouraging him from considering it. More broadly, several boys reported hearing similar remarks, and one 14-year-old male student explained how such messages shape perceptions among boys: “*If you tell a boy ‘this is a vaccine for girls,’ he’ll say ‘I don’t need it,’ and that’s that*” (Interview 43). These accounts show how gendered labeling of the HPV vaccine operates as a social shortcut that legitimizes disengagement among boys and perpetuates their exclusion from prevention efforts. However, this gender stereotype was challenged by some participants who recognized that “*boys… can also get the virus*” and transmit it (Interview 43). A vaccinated student emphasized: “*It’s not a girls’ thing. It’s for everyone*” (Interview 46). Thus, a strong demand for clear, non-gendered information about HPV is evident. Even among those aware of the vaccine, many would like a more thorough explanation of “*what it really does*” (Interview 39), the duration of protection, potential side effects, and the rationale for offering it at an age when one does not yet feel directly at risk of sexually transmitted infections. In this regard, one student observed that he prefers to address his vaccinations in order of immediate risk and cannot understand why this vaccine is offered in middle school if the risks lie in the distant future: “*It’s like homework: I’d rather do my assignments due Monday before doing those due Wednesday. I focus on what could happen right away; everything in its time. If I focus on things for when I’m thirty… well, I don’t have time to do them now*” (Interview 50). This comparison illustrates a prioritization logic in which health decisions are organized around perceived immediacy, making a vaccine associated with distant risks harder to integrate into adolescents’ present concerns, even when its preventive value is acknowledged. Like parents, adolescents frequently referred to prevention and to the influence of trusted people, whether family members, physicians, or peers, when explaining their decision. The most frequently cited reason for accepting the HPV vaccine was long-term health protection. More than half of the students (8/15) explicitly mentioned preventing serious illnesses and cancers as their primary motivation. *“For me, it was to avoid getting diseases and cancers*,” explained one student regarding her own vaccination (Interview 12), while another noted the infection’s prevalence: “*It means it’s less likely to catch the disease, since I recall it’s actually quite common*” (Interview 31). A desire to get ahead of potential risk was often expressed: “*Might as well do it now and get it over with*,” said one student, finding it “more convenient” to be vaccinated immediately so she could “*be worry-free for several years afterward*” (Interview 31). Similarly, another student emphasized, “*It’s better to get vaccinated now than be afraid of cancer in twenty years*” (Interview 46). This argument of long-term benefit resonated especially with those who understood the ultimate purpose of the HPV vaccine. Another decisive factor was encouragement from one’s social circle. Twelve out of fifteen students reported that a positive recommendation from a parent, physician, or trusted peer influenced their decision. “*It’s true that I’m going to do it because I know they’re not saying it just to bother me or anything*,” explained one participant regarding her family’s opinion (Interview 31). In some cases, protection of others was a motivating concern: a 9th-grade student explained that she and her boyfriend both chose to get vaccinated “*for each other*” (Interview 46), emphasizing that “*boys transmit it, so they must take responsibility*” for preventing virus spread. Although this sense of collective responsibility was less common, it was present among the most well-informed students. Finally, trust in medicine and the perception of the vaccine as a “routine” procedure also facilitated acceptance. Being able to discuss the vaccine with a healthcare professional and receive clear answers to their questions (for example, about side effects) relieved their concerns and confirmed their decision to be vaccinated. Conversely, some students reported not pursuing the HPV vaccine because of parental discouragement: “*My mom once said that the vaccine they mentioned here ‘wasn’t for boys,’ or something like that […] But she didn’t really explain, she just said no*” (Interview 42).

Several misconceptions noted among adolescents, such as uncertainty about what HPV is or who the vaccine targets, were likewise present among teachers, albeit expressed more as partial or muddled knowledge than as complete unfamiliarity. Teachers’ understanding of HPV and its vaccine was heterogeneous and often fragmentary. Just over half of respondents admitted to having limited knowledge of HPV: 8 out of 15 teachers reported that they did not feel well informed on the subject. Some had only a vague awareness, without grasping the details. For instance, one teacher confessed, “*Well… I’ve heard of it, yes. I couldn’t explain it in detail, but I know it’s a vaccine mainly for adolescents. I believe it’s linked to a virus that can cause cancer? Something like that. I admit I mix it up with others—there are so many*” (Interview 40). This lack of knowledge also manifested in incomplete or erroneous conceptions: several teachers (5/15) believed that the HPV vaccine was intended solely for young girls, unaware that it is also recommended for boys: “*I don’t know whether this vaccine can be administered to boys*” (Interview 26). These findings indicate confusion regarding both the scope of HPV infection and the target population for vaccination. By contrast, seven out of fifteen teachers demonstrate a more precise understanding of HPV and its vaccine. These informed teachers can identify HPV as a sexually transmitted virus capable of causing precancerous lesions or cancers—particularly cervical cancer—and are aware of the existence of a preventive vaccine. As one participant put it, “*For me, I immediately think of cancer prevention, especially cervical cancer*” (Interview 2). Such remarks reflect a fairly clear grasp of the stakes surrounding the HPV vaccine. Nevertheless, even among those teachers who recognize the seriousness of HPV, few are fully versed in all the scientific details (e.g., effects) or in the specific details (e.g., side effects, immunization schedule, recommended age), which may limit their comfort in addressing the topic in a school context: “*Parents are very well-informed nowadays. It is important to provide answers, yet we lack sufficient data on the outcomes, particularly concerning long-term effects and possible side effects*” (Interview 22).

Teachers’ uneasiness contrasted with what pharmacists reported, namely that many families *appear*, from their perspective, to refer to HPV vaccination as something more familiar, more visible, and generally more reassuring. The majority of pharmacists (12/15) noted a recent positive evolution in perceptions of and demand for the HPV vaccine. This favorable trend is frequently attributed to public awareness campaigns and improved dissemination of information. As two pharmacists noted, “*In recent years, I feel we have been vaccinating more and more. I think there must have been a campaign because it seems to be increasingly common*” (Interview 58), “*Maybe a bit of awareness of the potential danger it can represent*” (Interview 54), “*I don’t know if it’s because of the vaccination campaigns in middle schools, or the fact that it’s been talked about more, but… more and more of them are now coming on their own to get vaccinated*” (Interview 61). This shift is mainly attributed to changing attitudes and growing trust, reinforced by a snowball effect of communication, prescriptions and informal discussions among friends and family: “*There’s greater trust from parents now compared to a few years ago. This trust is mainly due to the fact that doctors are prescribing more—or at the very least, encouraging parents much more than they did in the past. Communication campaigns also play a role. And then, at some point, at school, “my daughter’s or my son’s best friend got vaccinated. ‘Oh, why not my son then?’” And so, it creates a kind of snowball effect. I think it’s mainly parents’ perceptions that have changed. In any case, I believe there’s much less hesitancy now than a few years ago*” (Interview 52). The majority of pharmacists appear to consider that the concerns observed 15 years ago have progressively diminished over time: “*Let’s just say it was a time when this vaccine raised more concerns than others. And it’s true that… yes, I’ve noticed a clear improvement in how the vaccine is perceived now*” (Interview 59). However, some professionals (3/15) observed increased vaccine hesitancy following the COVID-19 pandemic, pointing out that “*with COVID, people became significantly more reluctant. Many are getting fewer vaccines, especially when they are not mandatory*” (Interview 58).

### When prevention collides with fear, stigma, and institutional uncertainty

At the time of the interviews, only 2 out of 18 parents had not yet had their child vaccinated against HPV, citing ongoing concerns or reservations. The hesitations expressed fell into two main categories. First, both parents felt that the recommended age for vaccination was too young and were uncomfortable with the idea of addressing their child’s sexuality in that context. For example, one 43-year-old father explained: *“There’s also a sexual aspect to HPV, I think. And for my daughter, it just felt… a bit too early. She’s in eighth grade, but she’s born in December, so she still feels like a little girl to me. So yeah, I think it’s too soon”* (Interview 15). This account illustrates how parental discomfort with sexuality—combined with age and gendered representations of “innocence” and maturity—can shape perceptions of vaccine timing, potentially delaying uptake despite public health recommendations aimed at vaccinating prior to exposure. Second, concerns about potential side effects or a perceived lack of long-term data—classic features of vaccine hesitancy—were also raised by these same two parents. These worries, however, were rare among participants. Only 2 out of 18 parents expressed such concerns explicitly. Another parent, who did vaccinate their child, mentioned having initially wondered about potential side effects, but explained that hearing from acquaintances whose children had experienced no adverse effects was reassuring:*“I had already known two people—one whose son was vaccinated, and one whose daughter was—and their children didn’t have any symptoms. No side effects at all” (Interview 7).* Two parents also mentioned having come across false or conspiratorial claims—such as the idea that children might be “microchipped” through vaccination—without endorsing these views themselves. These comments reflect the types of misinformation circulating during the COVID-19 pandemic that parents may encounter, although such narratives did not appear to significantly influence most participants. These doubts were sometimes expressed directly by the parents, but more often came up in reference to their peers. One mother who had spoken with other parents reported: *“Some were a bit worried—concerned about side effects, and about whether the vaccine is really effective”* (Interview 3). She added that others were generally opposed to vaccination (“vaccine-hesitant”), whereas most people she knew believed that *“it’s a matter of health, so it’s extremely important. And anyway, it’s about our children’s future”* (Interview 3), emphasizing the predominance of a favorable attitude despite the existence of some reservations. In certain cases, access to better information helped overcome initial doubts. One mother explained that she had had *“some hesitation for myself at first,”* but that after *“looking things up in various places,”* she found enough reassuring information. By the time she had to make the decision for her daughter, she *“no longer had any particular reservations”* (Interview 3).

Hesitations were far more prevalent among adolescents, reflecting not only concerns about safety and long-term effects but also fear of the injection, discomfort discussing sexuality, and family constraints. Approximately half of the students (7/15) expressed hesitation or outright refusal of the HPV vaccine. The most common concern was fear of the needle and pain: about two-thirds of the students (9/15) spontaneously reported being afraid of the shot or the discomfort it might cause. As one 8th-grader put it, “*I was scared because when you talk about a vaccine, you think mostly of the needle… so, well, that’s scary”* (Interview 32). Many described the experience as unpleasant but brief and tolerable; this initial anxiety often eased afterward—*“in the end it was nothing big*” (Interview 32)—yet anticipation of pain remains a significant stressor. Reassurances from peers (“*they told me, ‘Don’t worry, it’ll be fine*’”; Interview 30) can mitigate this fear, but it nonetheless serves as a notable initial barrier. Furthermore, trust in vaccines varied. A large proportion voiced confidence when vaccination was recommended by trusted adults: “*If an adult—and especially a doctor or health professional—says it, then it must be respected, because they wouldn’t say that at random,*” stated one student, adding that if advised to vaccinate, “*I won’t argue*” (Interview 31). This deference to medical and parental authority underpins much acceptance. Conversely, another form of hesitation related to uncertainty about the vaccine’s safety or long-term effects also emerged: roughly one-quarter of students (4/15) expressed mistrust in this regard. Some demanded guarantees before consenting, not wishing to serve as “guinea pigs”: “*I want to see the effects on others first. If in five years everyone’s fine, okay, we can talk again. I’m not a guinea pig*” (Interview 44). They feared serious adverse effects—or even unfounded consequences such as infertility, a rumor one student reported hearing (Interview 39). These anxieties were sometimes fueled by family members—one pupil recounted, “*My dad… said you have to be careful*” with certain vaccines (Interview 42), citing limited hindsight on COVID-19 vaccines. Such mistrust reflects a craving for tangible data and scientific reassurance. Finally, embarrassment and taboos surrounding sexual health prevented some students from seeking information or vaccination. Several girls admitted feeling uneasy discussing sexual relations at home—“*we don’t really talk about those things*” (Interview 45)—and hesitated to ask questions in biology class for fear of judgment (“*I didn’t dare. I was afraid people would look at me. I preferred to stay silent*,” said a 13-year-old girl, Interview 45), especially when the teacher’s explanations felt “*really awkward*” (Interview 45). The very idea of requesting parental consent for a vaccine linked to sexual activity could be prohibitive: “*I wouldn’t really want to ask [my parents], and I think they’d be angry if I brought it up*” (Interview 45). These accounts suggest that anticipated judgment and moral sanction discourage adolescents from seeking information, reinforcing silence around HPV vaccination and limiting informed decision-making. This sexual taboo represents a barrier unique to HPV, not seen with more “routine” vaccines. Moreover, in two cases (2/15), parental opposition alone—parents deeming the vaccine “pointless” or preferring to have it administered later in a medical office—resulted in the adolescent not receiving the vaccine (Interview 42).

Among teachers, the obstacles took a somewhat different form: while some also reported discomfort around discussing sexuality, their concerns were largely shaped by professional constraints—limited information, lack of training, and fear of parents’ reactions. Teachers identified several major obstacles hindering both awareness-raising and the implementation of HPV vaccination initiatives within schools. The first obstacle, explicitly mentioned by 10 out of 15 teachers, is the lack of information and training among teachers themselves: “*Honestly, I don’t think I would be offered any training, as this topic does not relate to the subject I teach […] The Ministry of Education is increasingly reluctant to provide us with training, as it requires taking us away from our students, covering travel expenses, and ultimately represents a significant cost*” (Interview 26). Many reported feeling insufficiently informed about HPV or its vaccine to discuss the topic confidently with students. This knowledge gap fosters hesitation to bring up the subject, for fear of not finding the appropriate words or of unintentionally conveying inaccurate information: “*Situations like these always make me a bit uncomfortable, because I don’t have the appropriate tools to respond*” (Interview 41). Those with knowledge, particularly science teachers, acknowledge that it comes from their own research and that they do not receive information on HPV from their school: “*It’s just things we do personally. No—nothing at work at all. I, for one, wasn’t even aware of the vaccination campaign for students that took place about three weeks ago at my school*” (Interview 19). Moreover, the sensitive nature of sexuality inherent to HPV transmission was noted by 5 out of 15 teachers: because HPV is sexually transmitted, the subject is perceived as delicate to address in the classroom. As one teacher explained, “*Yes, I think there is real discomfort around this. As soon as we address sexuality in biology class or during a health education session, there’s giggling, whispering… it’s not easy to establish a serious atmosphere*” (Interview 51). Such discomfort reflects institutional and cultural constraints that limit the school’s capacity to address sexual health topics openly, thereby weakening the preventive potential of classroom-based HPV education. This unease, linked to the intimate character of the topic, can discourage some teachers from initiating dialogue—particularly in the absence of a clearly defined framework for discussion. Another significant obstacle pertains to fears of parental reactions, as noted by 8 out of 15 teachers. Many educators worry that some parents will view the school’s involvement unfavorably, considering it a matter confined to the private or family sphere: “*It’s complicated because I’m afraid of saying something that could backfire on me or the institution […] Otherwise, we take risks—and I’m also referring to parents’ reactions, which can be strong when they feel we have ‘overstepped our role’*” (Interview 41), illustrating the concern over potential conflicts with families. This apprehension can impede initiatives, since teachers fear being accused of overstepping their role or influencing students on a controversial subject without parental consent. Teachers seem to experience particular difficulties in their relationships with parents: “*HPV vaccination is around age 11 or 12, right? That’s when it’s up to the parents. So we actually need to target the parents too. But then… what do you do to get parents—who are getting dumber and dumber*^5^*—to see reason? I don’t know”* (Interview 4). Finally, a few teachers cited organizational or institutional constraints among the barriers: the lack of clear guidance from the Ministry of Education and the absence of dedicated time in the curriculum for such preventive actions can make it difficult to integrate HPV-related content into regular classroom activities: “*But it needs to be structured—not just a teacher mentioning something quickly during a lesson. There should be dedicated times, appropriate materials, and perhaps interventions by nurses or doctors. I’m willing to talk about it, but not on my own, and not without a clear framework*” (Interview 38). Teachers’ reluctance thus stems less from doubts about HPV vaccination than from uncertainty about institutional boundaries, particularly when health communication intersects with sexuality and parental authority.

Pharmacists, in turn, described concerns of a different kind centered not on institutional constraints, but on patients’ fears, misunderstandings, and practical questions about the vaccine. Pharmacists report that the most frequently expressed concerns among patients relate to the side effects of the HPV vaccine. Eleven out of fifteen participants regularly mention the need to reassure patients on this topic: “*The main concern is always the side effects […] They’re worried about whether a virus is going to be injected into them*” (Interview 55). While parents primarily ask questions about side effects, pharmacists report that children are more concerned about the pain of the injection: “*And from the children’s perspective, when I ask them, “Do you have any questions? Does it hurt?”—well, for them, the main concern is, “Is it going to hurt?”*” (Interview 53). Occasionally, these concerns are heightened by media events: “*With the story of the little boy who died after the vaccine, it got complicated*” (Interview 53), “*Parents and teenagers are aware that… well, during mass vaccination campaigns in middle and high schools, there have occasionally been cases where some adolescents fainted during or after the injection, or found the injection potentially painful*” (Interview 64). Pharmacists’ accounts highlight a split in concerns according to age, with parents focusing on safety and long-term effects, while children primarily express fear of pain, requiring different reassurance strategies within the same interaction. In addition, pharmacists also reported that some patients express confusion about the need to vaccinate boys, paraphrasing the comments they hear from parents: “*Why would we vaccinate my son against HPV? The vaccine is to avoid getting the virus and for uterine cancers—my son has nothing to do with that*” reported a 35-year-old male pharmacist (Interview 60), which pharmacists attribute to the initial communication efforts around HPV vaccination in France: “*In a completely irrational way, the State promoted vaccination for girls more than for boys. The recommendation to vaccinate boys—and even reimbursement—are very recent*” (Interview 52). Finally, several pharmacists report that parents often seek clarification about what exactly the vaccine will protect their children against: “*The primary purpose of this vaccination: sometimes, they’re not really aware of it*” (Interview 66). They also have questions about the vaccination schedule, including the number of required doses and the recommended timing for each: “*It’s really: “So, what exactly will this protect against?” That’s when we take a moment to explain. And especially: “Please repeat the vaccination schedule so I don’t forget. Tell me the dates—go over everything again”*” (Interview 53), “*The first question is usually: how does it work? Meaning, how many doses, at what age, is it reimbursed, can I get it at the pharmacy, how does it all go…*” (Interview 57). Alongside these day-to-day concerns, pharmacists identified a second layer of obstacles—more structural, attitudinal, or contextual—that also shapes whether families choose to vaccinate. Pharmacists identify three main barriers to HPV vaccination: the trivialization of HPV-related risks, resistance linked to vaccine-related beliefs or attitudes, and logistical obstacles. The tendency to downplay the seriousness of HPV is mentioned by ten pharmacists (10/15), as one participant explains: “*HPV is seen, collectively, as something benign*” (Interview 60). Nine pharmacists (9/15) report resistance rooted in a general mistrust of vaccines, often linked to past controversies within the French vaccination context: “*We still see a lot of hesitancy, stemming from the 1990 s, with concerns about autoimmune diseases linked to vaccines—especially multiple sclerosis, which scares everyone and was associated with hepatitis B vaccines. Basically, parents want to make sure that if their children are vaccinated, such incidents don’t happen again*” (Interview 52). These resistances seem amplified by post-COVID-19 anti-vaccine attitudes and rejection of non-mandatory vaccinations: “*COVID really held them back… it significantly discouraged them. So many people are now getting fewer vaccines, especially when they’re not mandatory*” (Interview 58), “*It’s strongly recommended, but it’s not mandatory. Some people, yes, will choose to ignore it*” (Interview 57). However, pharmacists appear to hold differing views on the impact of the COVID-19 pandemic, with some suggesting it may have contributed to a lasting increase in vaccination uptake: “*I really feel that COVID had a beneficial effect on vaccination. It really encouraged people to get vaccinated and to update their vaccination records*” (Interview 59). Finally, seven out of fifteen pharmacists highlight supply difficulties or the complexity of scheduling medical appointments: “*Given the rising demand for this vaccine in France—and globally, where demand is surging—I think we may face supply issues because of that as well. And I’ve already experienced some minor shortages in recent weeks.*” (Interview 52), “*I know we’re having quite a few supply issues. It’s not a major barrier, but we have to order in small quantities—sometimes we’re out of stock, sometimes we get just one dose, and other times we have three, four, even five vaccinations in a single day*” (Interview 63). Pharmacists also identify secondary barriers, such as the need for parental consent (“*The first obstacle is perhaps when a parent doesn’t want to have their child vaccinated*,” Interview 52) or situations involving parental disagreement (“*I’ve had several cases where the child or adolescent was actually the one asking for the vaccine […] It’s always a bit frustrating to see that—even though I’m not sure “convincing” is the right word—we’re unable to get the parent to agree and to help them see the importance of this vaccine*,” Interview 62) or divorce (“*I’ve also had cases—and I find these more problematic—where separated parents disagree: one wants to vaccinate the child, and the other does not*,” Interview 62). Vaccine hesitancy is also fueled by the perception that HPV vaccination is not urgent and that sexuality does not yet concern children—an important remaining barrier to uptake: “*It’s hard to say: “Be careful, children can be early, they might not talk to you about it” […] You can still sense that parents struggle to understand that even if the vaccine is given right at age 11, it doesn’t mean that getting the vaccine will lead their child to become sexually active immediately*” (Interview 53). These views are sometimes accompanied by beliefs about their children’s future sexual behavior—and that of their future partners—which are used to justify the decision not to vaccinate: “*Because as soon as she brings it up with young girls, the reaction is: “Oh no, my daughter isn’t like that, my daughter’s not a…” You see? And it’s true that this popular belief was very common at first. Less so now, but it still persists among people for whom religion plays a strong role*” (Interview 53). These statements illustrate a moralized interpretation of HPV vaccination, where preventive health actions are conflated with assumptions about sexual behavior, reinforcing stigma and delaying acceptance. Finally, two pharmacists point to differences in understanding and interest in HPV vaccination based on socio-economic status and suggest implementing targeted actions to address them: “*And it’s true that it’s the people who are more… more well-off, with a higher standard of living, who vaccinate their children more easily. Maybe because they’re also more informed. People coming from less affluent areas… it’s true that they tend to hesitate a bit more*” (Interview 61), “*I work in […]—it’s a wealthy neighborhood, and people are generally quite well-informed… it tends to go well. […] However, in areas where discussing sex and sexual relationships is more sensitive, I believe there’s important work to be done. It’s really about explaining the importance of getting vaccinated before the first sexual activity*” (Interview 63). Finally, pharmacists mention anti-vaccine individuals as a population they consider unlikely to be convinced: “*Some people are opposed to vaccination in general—it’s very difficult to change their minds. There’s not much we can do, other than provide them with general information*” (Interview 60), “*We have some among our clients—I know a few myself. And there’s a lot of misinformation, on social media, on YouTube, *etc*., which really acts as a barrier to our work*” (Interview 66).

### Trust, proximity, and legitimacy as key levers for vaccine acceptance

All parents expressed support for the involvement of schools as a channel for information and prevention regarding HPV vaccination. Nearly all of them (17 out of 18) considered middle school a relevant setting for raising awareness among adolescents and, in some cases, even for organizing the vaccination itself within the school environment. Several noted that schools are *“closer to the children than doctors are”* (Interview 15), and therefore can play a valuable complementary role—especially for reaching families who are not in regular contact with the healthcare system. Many stated that, had it not been for the campaign offered at school, they likely would not have taken the initiative themselves. *“I think I would have missed the information,”* admitted one mother regarding the vaccine (Interview 2). For some parents who were not well informed about the subject, the information session organized by the school nurse or the consent form to authorize vaccination served as a trigger. One mother, who was initially very skeptical, explained that being invited to a school meeting changed her mind: whereas she had previously been “categorically” opposed to vaccinating her son, *“he ended up doing it”* after the information provided *“freed her from her doubts”* (Interview 24). However, these sessions did not always provide sufficient information on their own: *“The school nurse held a meeting, but it was very limited—right before parent-teacher conferences. So I won’t lie: I had to do my own research, on different websites and through programs that covered the vaccine directly”* (Interview 1). Some parents highlighted the usefulness of these campaigns particularly for boys: *“I think it’s really helpful, especially for boys. Because people think more easily about vaccinating girls. And the mothers of boys might not think of it spontaneously. You don’t often go to the doctor with your son to talk about future gynecological problems, especially when it’s a boy”* (Interview 5). Another participant pointed out that stereotypes surrounding HPV vaccination for boys could be counteracted with more targeted communication: *“There should be two separate campaigns: one for girls and one for boys. Like, ‘Just because you’re getting vaccinated doesn’t mean you’re gay’”* (Interview 17). This quote highlights how HPV vaccination may become entangled with heteronormative assumptions and sexual identity-related stigma, suggesting that messaging may need to explicitly address and correct these misconceptions, while avoiding framing that inadvertently reinforces the association between vaccination and sexual orientation. This participant also stressed the importance of offering a discreet format to reduce the risk of stigmatization: *“You know, if a girl gets vaccinated at school, people might say, ‘She’s sleeping around.’ There’s going to be a negative spin. We need a discreet solution so they can get vaccinated without their friends knowing”* (Interview 17). This reflects the persistence of gendered moral judgments linking HPV vaccination to assumptions about female sexual behavior, which may discourage uptake and reinforce stigma. For 3 parents out of 18, the vaccination campaign offered in the seventh grade was a decisive factor in their decision. One parent emphasized the pedagogical effort put into the campaign: *“The school nurse would visit classrooms. When possible, she came during science classes, because teachers could then link it to their subject. Otherwise, she went into the homeroom periods, so not necessarily science. And there was time set aside for it—each class got about fifteen to twenty minutes”* (Interview 5). The logistical support provided (free vaccination on-site) also helped remove practical barriers, making it easier for many families to follow through: *“We had a form to fill out, I think. And then they could get vaccinated. I think it was about 15 days later, something like that. There was a waiting period. We had time to return the form, whether we agreed or not, and then the school coordinated with the nurse to do the vaccination. So it wasn’t a hassle for parents—we just had to say yes or no”* (Interview 5). Feedback on these school-based campaigns was overwhelmingly positive: parents described them as well-organized and *“smooth,”* saying that the process greatly facilitated their involvement—particularly with regard to managing multiple doses: *“The first dose is easy, but people are more likely to forget the second one. So I think if it had been done through the school, we wouldn’t have had to deal with it. I have four kids, so taking them all back to the doctor, scheduling appointments again, and so on—it would have been much easier that way”* said a 38-year-old mother (Interview 7). This success led some participants to suggest that such initiatives should be repeated and, if possible, extended to reach a wider population of students. As such, parents’ strong support for school involvement reflects less a transfer of medical authority than a pragmatic expectation that schools can lower informational and logistical thresholds, particularly for families less connected to routine healthcare pathways. Nonetheless, despite these very positive outcomes, some parents pointed to limitations in the school-based approach. The main issue cited was the lack of dedicated resources and uneven levels of engagement between schools. While some campaigns were described as very well organized, others required parents themselves to follow up with schools: *“For my second daughter, who’s in seventh grade, we heard about the school option through the media first, then through the school. But the information that came from the school was pretty limited. It’s a good thing we already knew what it was and were on board, because it was more like a basic notice—not really detailed”* (Interview 8). Several parents also noted that school nurses are often too few in number to conduct large-scale public health initiatives: *“Or maybe, if there is a nurse, she could just give a five-minute talk in each class, saying, ‘You can get vaccinated for free. If you want more information, come see me or…’ But you don’t find that in schools”* (Interview 17). Other parents suggested broadening the campaign to include additional grade levels (not just seventh grade), or renewing the messaging throughout the school years in order to reach adolescents before they lose interest in the subject as they grow older: *“It’s a shame to start only in seventh grade. I mean, maybe that’s when the recommended age begins, but they should just do it in all three years of middle school right away”* (Interview 2). Overall, schools were widely perceived as essential platforms for broad awareness campaigns—provided they are given the necessary support.

From the adolescents’ point of view, however, the main source of guidance about vaccination is not the school but the family, with parents—and to a lesser extent general practitioners—playing the central role in shaping their understanding of the HPV vaccine. A very large majority of students (13/15) cited their family—particularly their mother—as their main source of information about vaccines. “*Nearly everyone in my family was telling me it was better to do it*,” reported one student regarding the HPV vaccine (Interview 32), illustrating the strong impact of familial advice on decision-making. Family physicians also play a key role and are often mentioned alongside parents. Several pupils emphasized their trust in their general practitioner (GP): “*my doctor, I trust them; they’re clear and explain things well*” (Interview 43). Conversely, no adolescents recalled having discussed the HPV vaccine directly with a physician before receiving it, indicating that the initiative for information provision rests primarily with parents and, to a lesser extent, with school personnel. Approximately half of the students (8/15) reported that HPV vaccination had been addressed in some form at their middle school—either by a biology teacher or the school nurse. In some cases, this took the shape of an on-site vaccination campaign in year 7, with parental consent forms distributed and the school nurse’s involvement: “*The nurse came through the classes to say that if we wanted, she could give us the vaccine. That it was good, since sometimes parents struggle to get doctor appointments, and so she said it was great if we wanted to do it and that we could get vaccinated right in the nurse’s office*” (Interview 32). A few pupils recalled being vaccinated in small groups during class time: “*I was vaccinated at school. They called us out during lessons; there were several of us, and one by one we went to the room to get the injection […] They asked the parents, and the parents had to consent or not. If they agreed, they really did call us by groups of ten students.*” (Interview 30). Others remembered seeing informational posters in the corridors, receiving flyers (“*[the nurse] handed them directly to our head teacher, and she gave us the papers. She read through them with us a bit*”; Interview 33), or attending a biology lesson on papillomavirus as part of sex-education. However, many felt the topic was treated too briefly or superficially: “*In biology class it’s a bit rushed, and we avoid words like ‘sex,’ even though that’s the subject*” (Interview 46). Some did not take advantage of the few informational opportunities—out of shame or shyness they did not dare ask questions in biology class or found the nurse *“too awkward and uninviting to approach. It would be good to have a real session to talk about it without taboo. Because right now, some don’t dare ask their questions. I know—my friends asked me things because they didn’t dare, which is silly*” (Interview 46). Others may simply have been absent during the nurse’s visit (“*No one told me that. But maybe I wasn’t there*”; Interview 47). Thus, from adolescents’ perspective, the school emerges as a visible but incomplete source of information, offering exposure to HPV vaccination without consistently providing the depth or conditions needed for open discussion. Overall, the school’s role in providing vaccination information was perceived as insufficient by a majority of pupils. Many adolescents expressed a strong wish to receive more comprehensive information about HPV vaccination at school. Twelve out of fifteen students would like their middle school to allocate dedicated time specifically for HPV vaccine education. The preferred format for this information, however, varied. A majority emphasized the need for age-appropriate and discreet formats: they advocated for dedicated sessions—potentially in small, single-gender groups—where questions could be asked anonymously. One student suggested *“a session where we can submit questions anonymously, with a box*” (Interview 43), allowing sensitive topics to be addressed without exposing oneself before the entire class. The choice of facilitator also mattered. Several female students would prefer to speak with the school nurse “*because she’s experienced, it’s more discreet, and she’s here at school. She’s kind*” (Interview 45), rather than with a teacher whom they viewed as less legitimate or more distant in age and authority. Some boys, open to dialogue, believed that a younger educator or an external facilitator might be more effective in encouraging open discussion: “*If it’s a teacher who doesn’t really know the subject, sometimes they avoid it. So we don’t get enough information to really understand, and not enough people dare to talk or ask questions. Maybe a day with supervisors, because we can talk to them—they’re young and closer to us*” (Interview 43). The challenge identified by many is therefore to combat shame and ignorance: “*I would say that you shouldn’t be ashamed to ask questions…*” (Interview 46), advises a vaccinated pupil to her classmates, particularly the boys, who are often uncomfortable whenever sexuality is discussed. A minority of students (5/15) identified the media and social networks as channels for HPV vaccine information. For instance, one pupil learned about the HPV vaccine by watching television (“*TF1, on the evening news*”; Interview 22), while another encountered content online—sometimes alarmist or unreliable, such as TikTok videos. One student observed that interactive formats might engage him more than a traditional lesson: “*But if it’s a funny video or a game, I’m in. Otherwise, honestly, I don’t really listen*” (Interview 42). However, a female student noted that most social‐media resources seem aimed at girls: “*There’s tons of content for girls on social networks, but it looks like boys don’t watch that at all, which is a shame*” (Interview 46). In contrast, peer‐to‐peer discussion about vaccines was rare: only 3 of 15 students (3/15) reported actually talking with friends about vaccination outside the immediate context of getting the shot. Conversations among friends typically consist of brief comparisons of their experiences, rather than in-depth discussion. Some mentioned that male peers often make jokes—“*it’s for sex stuff*” or “*it’s useless*”—indicating the topic is more likely to be ridiculed than seriously debated (Interview 43). Nonetheless, in a few individual cases, friends who had been vaccinated provided reassurance and served as positive role models. One teenager recounted that “*almost all of my friends*” had already received the vaccine and assured her “*that it didn’t hurt, that it was nothing big*” (Interview 32), which prompted her to go ahead without further hesitation. Overall, though, vaccination remains a medical and intimate subject (particularly for HPV), which appears to inhibit spontaneous peer dialogue.

Taken together, these accounts show that adolescents navigate HPV information across several spheres—primarily within the family, and more marginally through school, media, and peers. Teachers, in turn, reflected on how the school should position itself within this broader constellation of actors and what role it can realistically play in HPV prevention and vaccination. When asked about it, teachers were divided, although most agreed that the school has an important part to play. For roughly two-thirds of them (10/15), the school should help inform and raise students’ awareness on health topics such as HPV vaccination: “*And since school is often the only shared space for all, I believe we should take on a more active role*” (Interview 41). They felt that, because of their close contact with adolescents and their parents, schools are ideally positioned to disseminate preventive knowledge: “*If you trust the teacher, if you feel that what they say… if they respect your beliefs, your whatever, *etc*., for many things, many other subjects, then they’ll trust us on certain stuff. And vaccination is one of those things*” (Interview 19). This majority of teachers saw the school as a complement to parents and healthcare professionals, particularly by providing reliable scientific information and encouraging students to protect themselves: “*These are sometimes topics that may not be addressed within families—due to a lack of knowledge, a certain sense of modesty, or perhaps a lack of engagement on the part of some families. In this context, yes, I believe that high schools have a definite role to play*” (Interview 26). A few even suggested that the school setting could host interventions by health professionals (e.g., doctors, school nurses) or vaccination campaigns, provided there is proper coordination with families: “*I think that interventions involving the school nurse, or perhaps associations invited to the school, might be more effective*” (Interview 26). One teacher suggested that, since schools already function as civic spaces—such as serving as polling stations during elections—it would be a logical extension for them to also be used as vaccination centers: “*Because schools are also often used as polling stations, there may be an opportunity to further develop the idea of setting up vaccination centers within schools—much like how they are used for voting*” (Interview 51). By contrast, a significant subset of teachers (5/15) expressed reservations about the school’s involvement in HPV vaccination. For these teachers, vaccination is primarily a parental and medical responsibility, and they believe the school should not intervene for fear of contradicting family beliefs or taking on a role that does not belong to it. As one teacher emphasized, “*So it should be up to doctors—when you go for your annual check‐up with your general practitioner—to address this topic, not the school […] Honestly, I don’t know if that’s my role*” (Interview 49). Others shared the feeling that they are not comfortable discussing such a personal issue with their students without proper training: “*Some colleagues still believe that it is only intended for girls, or that it is associated with early sexual activity—making it a taboo subject. It’s unfortunate, because as a result, the topic remains vague and sometimes somewhat uncomfortable to address*” (Interview 38). Overall, many stressed the need to establish a clear framework (i.e., parental consent, institutional support) before undertaking any HPV vaccination initiatives in the school setting: “*I’m not sure, because sexuality and religion are such difficult topics to address with young people these days… Honestly, I already have plenty of headaches at home; I wouldn’t really want to, quote‐unquote, put on myself more than what I already have to deal with every day*” (Interview 4). Despite the obstacles, teachers suggest various levers likely to facilitate acceptance and promotion of the HPV vaccine in the school context. A recurrent lever, proposed by 5 out of 15 teachers, is to involve health professionals in informational activities. Participants believe that inviting school nurses, physicians, or specialized external speakers to discuss HPV would lend scientific credibility and reassure both teachers and parents. As one noted, “*I believe that this communication should not fall solely on the teaching staff; it should also involve the medical community […] If we involve the medical profession, it will serve as an authority*” (Interview 37). Such an initiative would allow teachers not to shoulder the medical discourse alone and to respond more effectively to detailed questions from students or families. In this regard, one teacher noted that the organized interventions help to structure this type of process effectively: “*An intervention was carried out in each class by two healthcare professionals to provide information—first about what the HPV vaccine is, and then about how the vaccination campaign would be organized. It was clearly presented as voluntary, ultimately depending on the parents’—and to some extent the children’s—consent, but primarily the parents’*” (Interview 29). More generally, teachers feel that doctors can have a significant impact, particularly through the government-funded health check-ups offered free of charge at certain ages, such as those available for dental care: “*Or else, couldn’t we do something like… you know, like those dentist appointments for kids? […] A free appointment with the doctor to talk about HPV, you know*” (Interview 4). They found organizing these appointments at the pharmacy all the more interesting: “*If it could be handled by the pharmacist, in my opinion, it would be highly relevant*” (Interview 4). Another facilitator mentioned by several interviewees was the need for tailored, official educational materials. Four out of fifteen teachers stressed the importance of brochures, teaching kits, or ready-to-use content on HPV, validated by health authorities, to distribute or integrate into lessons: “*Something concrete: what it is, what it’s for, who it concerns, what the side effects are… And also: how should we talk about it with students? What words should we use? What could be misinterpreted?*” (Interview 40). One teacher also suggested creating a digital platform where students could seek reliable answers: “*Perhaps a digital platform where students with questions or concerns could directly connect with professionals to ask their questions*” (Interview 28). Improved institutional communication was also seen as a key facilitating factor: “*Campaigns—yes, including audiovisual ones—should be conducted by the Ministry of Health […] It would be important, in my view, to re-establish a proper course of action and make the vaccine more accessible. There should also be improved communication from the Ministry of Health to the Ministry of Education*” (Interview 22). Others proposed making vaccination a school-wide project led by the nurse, relieving teachers while ensuring institutional involvement. This reflects teachers’ desire for coordinated action: “*First and foremost, real coordination is needed—not just scattered bits of information here and there. It should be planned at the level of the school as a whole, involving teachers, the school nurse, possibly the student affairs coordinator, and even external partners*” (Interview 41). They noted, however, that this would not be possible without greater recognition for nurses and the provision of substantial resources: “*I think that, to begin with, the role of nurses needs to be strengthened—their presence, their visibility. Because most children, most of the time, don’t even know who the nurse is […] if students had a better understanding of and more confidence in the nurse, she could have a greater impact in what she communicates to them”* (Interview 4).

While teachers mainly discussed the institutional conditions under which the school could contribute to HPV prevention, pharmacists described the concrete, day-to-day strategies they use to inform, reassure, and accompany families in their vaccination decisions. In response to patients’ concerns, most pharmacists (13/15) favor a communication approach that emphasizes the benefits of the vaccine in relation to its potential risks: “*I always remind them a bit about the risk–benefit balance […] Generally, they understand it well: the risk of developing cervical cancer is far greater than the risk of a serious side effect from the vaccine*” (Interview 55). Their main argument is based on the well-established efficacy of the vaccine in preventing HPV-related cancers. One pharmacist explains: “*It’s mainly about communicating the effectiveness of the vaccine, the different cancers it can prevent. That’s really what we need to focus on*” (Interview 58). Pharmacists, however, appear to prefer maintaining a neutral stance, aiming to avoid making patients feel guilty or defensive: “*But I don’t say to them, “If you don’t do it, you’re going to get this.” That sounds a bit threatening, and I don’t like that approach. I prefer to be more supportive—saying something like, “This is what the vaccine is for, and studies have shown that…” That’s it. After that, they either go along with it or they don’t, but I’m not going to force them*” (Interview 57). The trust-based relationship established with patients is also a key lever used by pharmacists to reassure patients (9/15): “*Many of them—by the time we get to talking about Gardasil—we’ve already seen them before for many other things. So they’re comfortable approaching us. Parents trust us because they’ve already trusted us in the past*” (Interview 52). This approach helps to gradually share information about HPV vaccination and gives parents time to reflect before returning for the vaccination: “*Because you can tell when people come back a month later… you can sense that they’ve thought it through, that they’ve taken time to process it […] And there are often clear differences between the first time we talk about it and the second or third time—and when the dose is actually administered*” (Interview 62). To encourage uptake of the HPV vaccine, pharmacists identify several effective levers. Nearly unanimously (14/15), they emphasize the value of direct access to the vaccine in pharmacies, explaining that this flexibility aligns well with their daily practice and with what they observe among patients: “*The fact that we have extensive opening hours and immediate availability is a significant benefit*” (Interview 60), “*In our case, we do it without appointments. So it really happens whenever the person wants […] That way, setting up an appointment isn’t a barrier—it’s not something that would hold things up*” (Interview 57). Clear and transparent communication about the medical benefits of the vaccine is also seen as a major lever—especially when focused on cancer prevention rather than sexually transmitted infections, as was more common in past campaigns (13/15): *“Let’s say that when you say ‘it protects against cancer,’ people are more inclined to go for it. Whereas STIs might be a bit less convincing*” (Interview 59). More broadly, pharmacists emphasize the importance of multiplying awareness channels to support their role in vaccination efforts, particularly for populations with limited awareness or low levels of engagement: “*But the main focus should be on reaching people who don’t come to us proactively. Advertising campaigns, awareness efforts, television… I don’t know… maybe even including a small flyer in students’ school notebooks—something like that, with a message saying: “Talk to your doctor or pharmacist for more information”*” (Interview 57). Finally, the trust-based relationship established between pharmacists and patients is also cited as a key factor that facilitates communication and persuasion (12/15): “*They tell us about their lives—really, there is a close connection between us and the patients. So I think that if they have concerns or questions, they don’t hesitate to bring them up*” (Interview 59), “*It’s often questions like: “What do you think about it?” I think that’s the question we get asked the most*” (Interview 62).

### From information gaps to demands for credible, emotionally attuned communication

In total, 10 out of 18 parents believed that current communication about HPV vaccination is inadequate. *“I think we don’t get enough information as parents”* (Interview 1). Many explained that they had to look for information themselves—on the internet, through printed materials, etc.—to complement the limited details they had received. They expressed a desire for broader public information campaigns. According to these parents, communication about the vaccine should be more factual and supported by objective data. In particular, many said they would like concrete figures on the risks associated with the virus and the benefits of vaccination. They felt that being able to quantify the danger would help them better understand what is at stake. As one father put it, there is a need for *“statistics, data. I like to analyze risk and consequences. What are the risks if we don’t do it? What are the consequences today? Statistically, how many people have been infected? How many had complications?”* This need for evidence-based communication was often paired with calls for more impactful messaging. Some suggested “shock” campaigns, similar to those used for tobacco prevention, using emotionally powerful imagery to raise awareness: *“There needs to be numbers. Like those shock campaigns you see for smoking, with pictures of people on ventilators”* (Interview 1). One mother also emphasized the importance of simple, accessible language: *“Yeah, just put it really simply—not using terms we’re not used to. We’re not in the medical field, so even if it’s simple for them, for us it’s not. All these acronyms like HPV—people don’t necessarily know them”* (Interview 23). Parents also expressed concerns about the logistics of obtaining prescriptions and organizing vaccine appointments. They hoped for more “user-friendly” communication methods: *“If the school hadn’t done it, it would have been nice to get a letter—but a paper letter at home, not an email, because emails get lost in the noise. A paper letter that explains everything, and maybe even includes the prescription directly, so we can go get the vaccine—something that makes it easier”* (Interview 2). Four parents mentioned that reminders and appointment facilitation tools could be managed by trusted public institutions such as Ameli [57], regional health agencies (ARS, [58]), or the Ministry of Health [59]: *“You know how they offer a free dental check-up for kids? Well, something similar for HPV—like a free consultation with the GP to explain it—could be a communication tool”* (Interview 1). In addition, 5 parents saw the child’s health record as a potential channel to inform and follow up on HPV vaccination: *“Maybe in the health records, by age group—like when a child enters middle school, around 10—they could list this among the recommended vaccines”* (Interview 5). At the same time, other parents emphasized the importance of real-life stories to help reassure families. Testimonials from “people like them” could help build trust and motivate action. One mother suggested: *“Testimonials, like from a mom and her daughter—that would be good. Just regular people like you and me, to reassure parents and reassure kids. To tell them it’s for their own good and that there are no side effects”* (Interview 3). The intergenerational aspect (a parent and their child) and real examples of people who benefited from vaccination were seen as effective ways to make the message more tangible. Likewise, several parents believed that stories from individuals who had experienced HPV-related diseases could have a strong impact. Hearing, for example, from a woman who had developed precancerous lesions but recovered thanks to early detection, helped reinforce the perception of the virus’ seriousness and the value of vaccination. *“Hearing from women who’ve had HPV—it influences decisions. I’m one of them. I was lucky: it was caught early. I just needed a conization*^6^*”* (Interview 5). Parents’ simultaneous calls for statistical data and personal testimonials indicate that reassurance is expected to operate on both cognitive and emotional levels, suggesting that information alone is insufficient without credible, relatable framing. Finally, regarding communication channels, parents suggested diversifying efforts to reach broader audiences. While the role of the family doctor and the school was considered central, other means could complement their efforts. Commonly suggested tools included print materials and posters: *“Email communication, posters in schools, in doctors’ waiting rooms—raising awareness that way, I think, is a good thing”* (Interview 20), as well as television for reaching parents: *“Why not do TV ads to raise awareness among younger people? Their kids don’t watch. But even if it just reaches the parents, that already helps, and then the parents can do their part”* (Interview 20). Ten parents also mentioned social media. Eight of them suggested using influencers followed by young people to communicate preventive messages in a way that resonates with adolescents: *“Social media, like Instagram. Influencers. If some influencers—maybe ones who had HPV or got vaccinated—talked about it openly, I think that would make it easier to reach teens”* (Interview 5). This strategy could help highlight reassuring data on the vaccine’s safety and efficacy. Some parents stressed the importance of relying on trusted influencers to reduce the risk of misinformation: *“There should be influencers—ones who are known to be serious—giving reliable information about this”* (Interview 20). Their goal was to normalize the HPV vaccine, make it more visible, and dispel common fears. In this regard, 6 out of 18 parents explicitly voiced concerns about disinformation related to vaccines or health more generally, particularly HPV. They emphasized the need to counter false information circulating online, including through trusted digital channels: *“It has to come through social media, but from a source you can trust. That’s what’s important”* (Interview 7).

These strong expectations for clearer, better-structured information were echoed—albeit from a different angle—by teachers, who emphasized that they too lack up-to-date knowledge, concrete pedagogical tools, and accessible materials to address HPV vaccination confidently with students and parents. Fourteen out of fifteen teachers stated that they would need more comprehensive, reliable, and up-to-date information on HPV and its vaccination. Many feel a lack of personal training on this specific health topic. As one teacher noted, “*Personally, I have never been consulted or trained on this topic in particular*” (Interview 51). Dedicated training—or at minimum an information session led by experts—is desired to deepen their knowledge and enable them to answer students’ or parents’ questions without hesitation: “*There should be extensive communication, including, if necessary, meetings with experts to ensure accurate information is conveyed. This is precisely what seems to be lacking*” (Interview 22), “*First of all, health-oriented training sessions would be useful. For example, having a medical team—such as a nurse or doctor—intervene to raise our awareness, remind us what HPV is, who can potentially be affected, and which populations should be targeted*” (Interview 37). Simultaneously, teachers express a need for concrete pedagogical resources to address HPV in class. Educational tools—such as slide presentations, age-appropriate videos, simplified information sheets, or brochures directed at students and their parents—are in high demand. As several teachers emphasized, “*we need concrete materials: posters, brochures, videos, and teaching resources tailored to each grade level*” (Interview 41), “*I also believe that a large-scale communication campaign is needed—whether through flyers, television, or televised news programs*” (Interview 37), highlighting the current lack of available materials. Other teachers noted the importance of explaining the risks of HPV and offering testimonials: “*I think the thing that will work best is to really talk about the risks and diseases one could contract, and how to avoid them […] I think it’s the numbers that can make an impact*” (Interview 9); “*And maybe testimonials from people who have had HPV—well, from women who have had HPV—testimonials from women who have had cervical cancer more or less related to it, and who come to explain…*” (Interview 4). In general, these resources would help structure the discourse, legitimize the teacher’s guidance, and ensure consistent information. They would also harmonize the messages conveyed across the institution, preventing each teacher from improvising in isolation, particularly science teachers during their lessons on sexuality: “*And when I cover condom use, I also mention that there are certain vaccines. I bring it up during my class… but it still stays within the framework of the curriculum*” (Interview 19). Therefore, teachers’ demand for ready-to-use materials reflects not only informational needs, but also a search for institutional backing that clarifies their role and legitimizes addressing a topic perceived as sensitive. To complement the teaching methods used in class, some teachers are emphasizing the value of social networks in providing young people with access to information: “*What is likely to truly resonate with young people is awareness-raising through social media—Snapchat, Instagram… platforms they use on a daily basis*” (Interview 37), *“Maybe if it was on TikTok, or… I think they also use Snapchat a lot. I don’t know… making videos to raise awareness. I imagine that already exists, I have no idea. And maybe if those videos were made by influencers… both female and male… that they follow*” (Interview 4). Although some questions the quality of the information provided, “*And social media don’t help either in that regard. Since anything can circulate, it creates a bit of stress*” (Interiew 9). These fears go hand in hand with an increased perception of the anti-vaccination movement since the COVID pandemic: “*And it is clear that the COVID vaccine has not helped the situation. There has been a great deal of speculation in that regard. In fact, it seems that the very term ‘vaccine’ has become the issue*” (Interview 28).

What teachers describe—above all, the need for clear, ready-to-use materials and expert support—mirrors the very challenges that pharmacists encounter when families eventually turn to them for answers. It is at this later stage, in direct contact with parents and adolescents, that pharmacists identify their own training and communication needs. While most of the pharmacists interviewed (14/15) feel sufficiently trained to answer patients’ common questions, they nevertheless express a need for regular, up-to-date training (“*What we retain after 7 days, 6 months, or a year… there’s always some loss of knowledge. So there’s always a need for ongoing training*,” Interview 52), as well as tailored educational materials to support counseling at the counter: “*It’s always helpful to have small posters, little cards, A5 formats summarizing the key information for patients*” (Interview 55), “*There’s so much information readily available online that we can also direct them—or redirect them—to those websites*” (Interview 52). These regular updates are particularly valued as an opportunity to clarify specific or atypical situations: “*We were outside the usual procedures, and I was able to discuss it with the representative. I found it very helpful that he came and provided a clear explanation*” (Interview 59). Some pharmacists report having received thorough training and comprehensive materials (“*I’ve also received additional training provided by the pharmaceutical company, which sometimes includes brief refreshers—phone calls or online sessions—that are fairly didactic, concise, and practical*,” Interview 60), while others mention not having access to such extensive resources (“*But beyond that, we’re not necessarily well trained—it’s true that we don’t always receive follow-up training from the pharmaceutical company, with representatives coming to provide us with a general overview of the vaccines*,” Interview 55). Those who have not received specific training would particularly like to receive information that enables them to address patients’ main concerns, especially regarding how the vaccination is organized and its potential effects: “*It shouldn’t be too long. It needs to be general—reminding us of the indications, contraindications, the vaccination schedule—and provide all the tools that would allow us to be 100% professional when it comes to prevention […] Every year, [a laboratory] gives us a little kit before flu season with flyers, small vaccination calendars, and reminders about the vaccines. That’s really helpful*” (Interview 54). Six out of fifteen pharmacists also suggest specific training in vaccine communication to better handle patient hesitancy: “*When it comes to discussing with patients, yes, it could be useful to focus on how to encourage vaccine adherence—perhaps through more concrete case examples […] To remind us, on the one hand, of the key points about the vaccine itself, and to perhaps provide us with tools to respond to patients’ concerns in the moment.*” (Interview 60). One participant particularly emphasizes the relevance of providing scripted dialogues to support the management of specific situations: “*It could be useful to have video-based information, perhaps showing real-life interaction scenarios—like a “counter situation,” so to speak—a video of a conversation, or even typical questions along with suggested responses to help guide the patient toward vaccination, or at least toward information that allows them to reflect on their own and understand the benefits of vaccination compared to not being vaccinated*” (Interview 60). Pharmacists’ repeated requests for concise and up-to-date materials indicate that their capacity to reassure families relies less on questioning their role than on having practical, reliable tools that support clear and efficient counseling in everyday interactions. Tables 2, 3, and 4 together synthesize the study’s insights by, first, mapping HPV-related perceptions, barriers, facilitators, and information needs across participant groups (Table 2), then comparing these groups to identify shared and divergent patterns (Table 3), and finally outlining implementation-oriented recommendations from a multi-stakeholder perspective to strengthen HPV vaccination in France (Table 4).

**Table 2:** Summary of HPV vaccination perceptions, knowledge, barriers, facilitating factors, and communication needs across parents, adolescents, teachers, and pharmacists

Themes	Parents	Adolescents	Teachers	Pharmacists
Vaccination as a normative yet unevenly embodied public health practice	Predominantly positive perception, trusting healthcare recommendations; some concerns about side effects and number of vaccinations for children (M)^a^	Generally positive perception, understanding preventive role; anxiety primarily about injection pain (M)	Generally positive perception, recognizing public health benefits; minority express concerns about side effects or insufficient long-term safety data (M)	Positive perception overall, emphasizing proactive educational role and trusted relationship with patients (F)
HPV vaccination caught between fragmented knowledge, gendered representations, and late awareness	Limited, inconsistent knowledge; misconceptions regarding gender applicability and transmission modes common (B)	Limited knowledge, frequent misconceptions about gender specificity and HPV transmission (B)	Variable knowledge levels; frequent misconceptions particularly about applicability for boys (B)	Strong knowledge, emphasizing cancer prevention efficacy; aware of misinformation and gender misconceptions (F)
When prevention collides with fear, stigma, and institutional uncertainty	Concerns about side effects, inappropriate age for vaccination, discomfort discussing sexuality with children (B)	Fear of injection pain, embarrassment discussing sexuality, concerns about vaccine safety and side effects (B)	Lack of detailed HPV information, discomfort discussing sexuality in class, fears of negative parental reactions (B)	Patient concerns predominantly about vaccine side effects and misinformation spread online (B)
Trust, proximity, and legitimacy as key levers for vaccine acceptance	Trust in healthcare professionals, proactive school vaccination campaigns, clear, factual communication (F)	Support from family, peers, healthcare professionals; clear understanding of vaccine’s long-term health benefits (F)	Schools as critical platforms for HPV vaccine information dissemination; involving healthcare professionals for reassurance (F)	Convenience and availability in pharmacies, trusted patient relationships, clear communication of vaccine efficacy (F)
From information gaps to demands for credible, emotionally attuned communication	Desire for evidence-based, accessible communication, real-life testimonials, expanded media involvement (F)	Clear, age-appropriate communication; discreet and anonymous school-based interventions; anonymous Q&A platforms (F)	Enhanced professional training, clear guidelines, dedicated educational materials (F)	Ongoing professional training, clear educational materials for effective patient communication (F)
Specific suggestions	Data-driven public health campaigns, targeted communication by trusted health institutions (*e.g.*, CPAM, ARS), integration into routine healthcare records (F)	Dedicated educational sessions, involvement of trusted social media influencers (F)	Collaboration with healthcare professionals, clear institutional guidelines, comprehensive support materials (F)	Increased visual aids, factual data presentations, specialized training to address vaccine hesitancy (F)

^a^Role codes: *(F)* facilitator, *(B)* barrier, *(M)* mixed

**Table 3:** Comparative analysis of similarities and differences between groups regarding HPV vaccination perceptions, knowledge, barriers, and information needs

Themes	Common points across groups	Divergent points among groups
Vaccination as a normative yet unevenly embodied public health practice	Overall positive perception, recognizing vaccines’ preventive health benefits (F)	Adolescents uniquely focused on anxiety related to injection pain; pharmacists specifically highlight proactive educational role (M)
HPV vaccination caught between fragmented knowledge, gendered representations, and late awareness	General lack of complete understanding about HPV vaccine, with frequent gender-related misconceptions (B)	Pharmacists display notably stronger knowledge of vaccine efficacy and HPV-related cancers than other groups (F)
When prevention collides with fear, stigma, and institutional uncertainty	Concerns about vaccine side effects and discomfort discussing sexual health issues (B)	Adolescents uniquely focused on embarrassment and injection pain; teachers notably concerned about parental reactions (B)
Trust, proximity, and legitimacy as key levers for vaccine acceptance	Trust in healthcare professionals and clear communication emphasized by all groups (F)	Pharmacists uniquely emphasize convenience and direct vaccination availability; teachers strongly value school’s role in information dissemination (F)
From information gaps to demands for credible, emotionally attuned communication	Clear, accessible, and factual information consistently desired (F)	Adolescents uniquely emphasize discreet, anonymous communication channels; parents particularly highlight real-life testimonials and broader media involvement (F)
Specific suggestions	Improved communication materials and involvement of healthcare professionals consistently recommended (F)	Adolescents propose involving social media influencers and anonymous platforms; pharmacists particularly suggest visual aids and specialized professional training (F)

^a^Role codes: *(F)* facilitator, *(B)* barrier, *(M)* mixed

**Table 4:** Implementation-oriented recommendations from a multi-stakeholder perspective to strengthen HPV vaccination in France

Recommended action	Stakeholders involved	Objective/rationale
Strengthen the school’s role in HPV information, with teacher toolkits co-developed with health professionals (e.g., ready-to-use slides, short videos, structured Q&A sheets) and institutional support	Ministry of Education, health authorities, school staff (i.e., teachers, school nurses)	To legitimize and normalize discussions about HPV vaccination in schools, reduce teachers’ discomfort, and ensure reliable, age-appropriate information
Provide regular training for school staff (e.g., workshops, continuing education modules) via partnerships between health and education authorities	Regional health agencies, education authorities, specialized trainers, teachers, school nurses	To increase teachers’ knowledge and confidence about HPV vaccination, addressing sensitive issues such as sexuality and side effects, and to institutionalize the school’s role in prevention
Integrate emotional dimensions into communication, using peer stories, testimonials, engaging visuals rather than factual messages alone	Public health agencies, communication specialists, patient associations, schools (i.e., for dissemination)	To address emotional barriers (e.g., fear of side effects, anxiety about injections, embarrassment about sexuality) that facts alone cannot overcome
Co-create information materials with adolescents, involving youth ambassadors and associations	Health authorities, schools, youth associations, adolescents	To ensure content is relevant, credible, and adapted to adolescent values, language, and concerns, while fostering ownership and empowerment
Develop social media campaigns with youth-created content, under expert supervision for accuracy	Ministries of Health/Education, regional health agencies, schools, youth groups, communication experts	To reach adolescents on platforms they use daily (e.g., TikTok, Snapchat, Instagram) with attractive content that remains scientifically accurate
Mobilize influencers on social media, especially micro-influencers known for educational/health content, equipped with validated information and visual assets	Public health agencies, micro-influencers, health communicators	To reach hard-to-reach adolescents who distrust traditional institutions, by using trusted digital voices
Implement an integrated school-pharmacy model: schools provide structured classroom sessions (e.g., anonymous Q&A, age-appropriate, non-gendered materials, visible role for the school nurse), while pharmacies ensure walk-in vaccination, supportive counseling, and reminders for the second dose	Schools (e.g., teachers, nurses, principals), pharmacists, local physicians, health authorities	To combine universal education with accessible vaccination, simplifying the vaccination journey and ensuring series completion
Ensure coordination and leadership, ideally by the school nurse, with planned activity cycles (e.g., one session per class per term), coordination with pharmacies, and equity monitoring dashboards	School nurses, school leadership, regional health agencies, pharmacists	To sustain implementation over time and guarantee that all eligible students are informed and followed up, while monitoring equity in coverage
Disseminate harmonized national messages (e.g., non-gendered, cancer-prevention frame) that teachers and health professionals can reuse verbatim	Ministries of Health and Education, national health agencies, professional associations	To ensure coherence and reduce message drift, avoiding contradictory or stigmatizing communication
Involve trusted healthcare professionals (e.g., GPs, pediatricians, midwives, school nurses, pharmacists) in systematic promotion and vaccination delivery	Primary care professionals, health authorities, professional associations	To leverage their credibility and trust relationships with families, addressing hesitancy and ensuring consistent promotion across all healthcare touchpoints

## Discussion

### Context and main results

Despite the availability of an effective, well-tolerated HPV vaccine, coverage in France remains low (e.g., 41.5% girls, 8.5% boys vaccinated at age 15 in 2021; [60]). Recent policies, such as school-based vaccination, aim to improve uptake yet continue to face major obstacles [23–26]. To understand these barriers, our qualitative study examines perceptions of adolescents, parents, teachers, and pharmacists regarding HPV vaccination.

As summarized in Tables 1, 2, and 3, our findings point to both shared and group-specific patterns of HPV vaccine hesitancy. While attitudes toward vaccination for cancer prevention were generally positive, participants across groups reported barriers related to fear of side effects, misinformation, and discomfort linked to the vaccine’s association with sexuality. These concerns were often emotionally driven among adolescents and parents, whereas teachers and pharmacists primarily emphasized structural constraints, such as limited training, unclear institutional roles, and insufficient support. Despite these barriers, schools were widely recognized as a potentially effective setting for HPV prevention, provided that adequate resources, training, and institutional backing are in place.

Taken together, these group-specific patterns reveal that emotional barriers (e.g., fear, embarrassment, stigma) dominated among adolescents and parents, whereas teachers and pharmacists primarily pointed to structural barriers, suggesting that interventions should differentiate between emotional determinants for families and institutional challenges for professionals. All groups called for clearer, more accessible, age-appropriate communication materials. Trust in healthcare providers emerged consistently as crucial, underscoring pharmacists’, GPs’, and school medical staff’s potential in overcoming hesitancy, provided they receive sufficient training and communication support. Notably, the figures of trust varied across groups: adolescents tended to rely on school-based health professionals, parents primarily trusted GPs, and pharmacists described themselves as accessible yet under-recognized sources of reassurance, highlighting that trust dynamics differ between populations and should be leveraged accordingly.

### Contributions of the study in light of the existing literature

Our findings confirm well-established barriers to HPV vaccination from previous studies and offer new insights via a multi-stakeholder perspective. Consistent with existing literature, fear of side effects notably drives vaccine hesitancy, particularly among parents and adolescents, highlighting the need for clear, reassuring communication [25, 28, 36]. Both pharmacists and teachers emphasized insufficient knowledge and tools, underlining the importance of dedicated resources—findings consistent with recent French research showing widespread gaps among school staff (e.g., fewer than half knew HPV could cause conditions other than cervical cancer; [14]).

Structural issues further exacerbate this gap; according to prior research, school nurses, despite viewing HPV education as their responsibility, rarely address the topic due to inadequate training and tools, compounded by their limited availability in schools [14]. Consequently, teachers often become primary information providers despite feeling unprepared and fearing parental backlash, with some fundamentally opposing school-based vaccination—findings consistent with prior research [14, 45]. In our study, teachers similarly reported feeling undertrained and uncertain about how to address HPV vaccination in class. Participants strongly recommended that healthcare professionals, especially GPs, lead vaccination promotion, believing this would enhance parental trust. Although literature highlights schools’ effectiveness in achieving high adolescent vaccine coverage and teachers’ potential advocacy roles [45], our findings indicate that the teachers we interviewed primarily viewed their role as informational rather than actively facilitating vaccination, often due to limited knowledge, skills, or perceived role boundaries shaped by disciplinary background. Science teachers showed more engagement but acted individually rather than through institutional support. Thus, clarifying teachers’ role through better coordination with healthcare professionals and targeted training appears crucial.

This ambiguity around teachers’ roles underscores the importance of considering multiple stakeholder perspectives simultaneously, as group-specific concerns can inform targeted strategies. In a recent qualitative study, Ailloud et al. [32] showed that mothers and educational staff expressed different types of concerns. Mothers focused on gynecological follow-up, expressed mistrust toward pharmaceutical companies, and questioned the reliability of online information. In contrast, educational staff emphasized cultural and religious influences, the role of local authorities, and the logistical difficulties of organizing school-based vaccination. Our study broadens this understanding by adding adolescents’ and pharmacists’ viewpoints. Adolescents notably expressed a strong desire for increased involvement and autonomy in vaccine-related decisions, aligning with previous findings by [61].

In addition to adolescents’ growing autonomy, pharmacists’ perspectives provide insight into how their preventive activities may intersect with efforts to improve HPV vaccination. Building on their expertise and patient trust, pharmacists described being open to taking on additional preventive activities, including vaccination, within the scope of their current practice—an approach particularly effective given parents’ reliance on pharmacist competence when choosing pharmacy-based vaccination [40]. This aligns with international evidence highlighting pharmacists’ accessibility, strong patient relationships, and vaccine delivery experience, while emphasizing the need for targeted training, tailored communication materials,^7^ and practical support (e.g., electronic reminders) to enhance their role effectively [40, 42, 62, 63]. Pharmacists in our study particularly stressed the importance of clear informational resources, consistent with evidence showing positive impacts on vaccination rates [63]. Given that pharmacists sometimes overestimate their HPV-related knowledge, continuous access to accurate information remains essential [42]. Structural supports, such as integrated software alerts for vaccine eligibility and dose reminders, were also strongly endorsed [42]. Moreover, our findings reaffirm frontline healthcare professionals’ crucial role, echoing GPs’ experiences of regularly addressing parental hesitations and misinformation related to side effects and sexuality concerns [23, 36]. Participants and prior literature suggest a complementary model: GPs initiating vaccination and providing initial counseling, while pharmacists manage follow-up doses. This approach could alleviate GPs’ heavy workloads—often limiting their HPV knowledge updates—and capitalize on pharmacists’ trusted patient relationships, particularly valuable amidst growing public mistrust toward some health authorities and physicians [25, 36]. More broadly, pharmacists’ routine involvement, currently expanding in France, could mitigate the vaccine hesitancy documented among many GPs [34].

### Integrated behavioral and implementation model

HPV vaccination behaviors across our samples can be best understood through the COM-B and CFIR frameworks. The COM-B model (i.e., Capability, Opportunity, Motivation—Behavior) conceptualizes behavior as the result of individuals’ psychological and physical capability, their social and physical opportunities, and their reflective and automatic motivations [64]. The CFIR (i.e., Consolidated Framework for Implementation Research) provides a comprehensive structure for analyzing how outer-setting signals, inner-setting readiness, characteristics of individuals, and implementation processes interact to shape the uptake of interventions [65]. Applied together, these frameworks illuminate that barriers to HPV vaccination lie less in general awareness gaps than in specific informational needs and in constraints in capability, opportunity, and motivation. Adolescents and teachers lacked targeted information on HPV (e.g., indications for boys, schedule), while pharmacists requested concise tools for counseling; physical capability was rarely limiting. Opportunity varied by context: pharmacies offered accessibility through walk-in services and extended hours, whereas schools provided broad reach but were constrained by time, privacy, and continuity. Social opportunity depended on trusted figures such as parents, pharmacists, and school nurses, yet was weakened by the sensitivity of discussing sexuality. Motivation combined reflective calculations (e.g., cancer-prevention benefits, strengthened by simple schedules) with automatic responses (e.g., needle fear, anticipatory anxiety), both strongly shaped by the credibility of messengers and the style of counseling. This mapping reveals misalignments between outer-setting signals (e.g., long-standing girl-focused communication), inner-setting readiness (e.g., limited training, unclear roles in schools), and weak processes (e.g., absence of structured sessions and systematic handoffs), which together explain why favorable attitudes often fail to yield completed vaccination series. Furthermore, stigma and sexuality-related concerns emerged strongly in our data, pointing to socio-emotional barriers that require more than capability, opportunity, and motivation-based adjustments.

Bridging these gaps requires targeting the psychological and social mechanisms that translate information into action. Reframing HPV vaccination as cancer prevention reduces stigma and resonates with parental identities whereas STI framing fuels discomfort [66]. Adolescents’ present-oriented reasoning highlights the need to reduce temporal distance, for instance by emphasizing immediate protection and enabling same-day vaccination [67, 68]. Norms and identity also play a decisive role: gendered stereotypes and peer ridicule undermine boys’ engagement, indicating that communication strategies must acknowledge, not erase, the role of gender [69]. Approaches that make boys’ involvement visible and use trusted messengers may help reduce stigma and support their sense of relevance in HPV vaccination [69]. These insights point to practical behavior change techniques such as reframing, prompts and cues, clear information on health consequences, and brief routines to reduce anxiety. Importantly, several of these concerns, those related to stigma and sexuality, are socio-emotional rather than informational, and thus cannot be fully addressed by capability or opportunity-focused adjustments alone. Recent evidence shows that such stigma-linked beliefs require complementary relational and psychological strategies, including strengthening parents’ communication self-efficacy and explicitly correcting the misconception that vaccination encourages sexual activity [70], as well as culturally sensitive, stigma-reducing messages that normalize sexual-health discussions [71]. Taken together, these insights highlight the need for implementation strategies that combine accurate information with relational, dialogue-based approaches capable of addressing socio-emotional barriers.

On this basis, the most promising implementation model is a school-pharmacy partnership in which schools create informed demand and pharmacies provide accessible, reliable completion. Concretely, this means short, scheduled classroom sessions with opportunities for anonymous questions, age-appropriate materials with an inclusive, non-gendered entry framing (as requested by many participants), complemented by gender-informed content when needed, and a visible coordinating role for the school nurse—with delivery led primarily by school nurses or trained external facilitators. Teachers would be expected to attend these sessions, while their level of active involvement (e.g., co-facilitation) would remain voluntary and based on individual comfort. Pharmacies would then ensure walk-in vaccination, brief supportive counseling, and automated reminders for the second dose. In parallel, given participants’ emphasis on the decisive influence of trusted healthcare professionals, general practitioners should be explicitly integrated as initiators of the vaccination conversation and aligned with school-based information and pharmacy-based access (e.g., triadic model: school-pharmacy-GP). Effective implementation further requires clear leadership (often the school nurse), planned activity cycles (e.g., one session per term), coordination with local pharmacies to track completion, and dashboards to monitor equity and progress. Finally, harmonized national messages that teachers and pharmacists can reuse verbatim would minimize message drift and cognitive load while anchoring initial communication in an inclusive, non-gendered cancer-prevention frame, while allowing tailored, gender-informed clarifications to address specific concerns. This integrated approach offers concrete directives to strengthen capability with targeted tools, expand opportunity through coordinated delivery, and sustain motivation via trusted, emotionally resonant communication.

The emotional dimensions of vaccine hesitancy warrant greater attention. Participants frequently cited fear of side effects, discomfort discussing sexuality, and injection anxiety—barriers that factual messages alone fail to overcome. While these concerns were often described as signs of growing hesitancy in our sample, national data suggest a different picture: vaccination adherence in France remains high and stable [72]. Rather than indicating a true rise in refusal, these expressions of doubt may reflect that individuals feel more comfortable voicing questions or uncertainties in the current context. However, our qualitative data do not allow us to determine whether this represents greater openness to discussion or merely a temporary amplification of concerns. Communication strategies must address these affective responses. Evidence shows that emotionally resonant formats—peer stories, testimonials, and engaging visuals—are more persuasive, particularly for adolescents and individuals with low health literacy [39, 73]. Videos appear especially effective: animations use vivid cues and debunking, story-based formats highlight disease severity, and velfies^8^ foster authenticity and identification, particularly among youth [74]. Tailoring such content to emotional and cognitive needs may boost campaign impact. Informational materials targeting adolescents could include concise, age-appropriate explanations of HPV transmission and cancer prevention, a clear breakdown of the vaccination schedule (i.e., number and timing of doses), and direct answers to common concerns—particularly regarding side effects, pain, and the relevance of vaccination before sexual debut [75]. Public health actors could invest in co-creating these materials with youth ambassadors, schools, and civil society groups, ensuring that formats and messaging resonate with adolescent values and language [75]. Ministries or regional actors might also pilot social media campaigns using youth-created content under expert supervision, ensuring both appeal and accuracy.

Moreover, this aligns with adolescents’ call for tailored information and active involvement, reinforcing the need to treat them as autonomous health actors rather than passive recipients, as previously highlighted [61]. Interactive school workshops, peer education programs, or participatory campaigns where students design awareness activities could provide meaningful engagement opportunities [75]. However, the convergence of perspectives across all four participant groups gave particular weight to a shared concern about the reliability of information on social media, echoing prior research reflecting adolescents’ doubts and mistrust toward online content [39]. Enhancing the quality and visibility of trustworthy content on these platforms is thus essential to reach youth often missed by traditional channels (e.g., healthcare providers). In response, participants in our sample and in previous studies recommended mobilizing influencers on platforms like Snapchat or Instagram—a promising strategy to build trust and visibility among hard-to-reach groups. This could be operationalized through partnerships with micro-influencers known for educational or health content [76], who would be provided with accurate, accessible HPV messaging and visual assets designed by public health agencies. Table 4 consolidates these cross-cutting findings into a set of concrete, stakeholder-specific recommendations, indicating who should act, how, and for what purpose.

### Limitations and future directions

Despite its valuable insights, this study has several limitations. First, as is typical for qualitative inquiry, our aim was not statistical generalization but analytical depth and transferability of insights. The small purposive sample means that the perspectives captured are necessarily situated, and some viewpoints may remain unrepresented. Participants—recruited from a few pharmacies and secondary schools—may not reflect France’s full socio-cultural diversity. Moreover, parents and adolescents who agreed to participate were likely more engaged with vaccination issues, potentially shaping their responses. Future studies should therefore aim for broader, more diverse samples across regions and social groups. Regional disparities in HPV coverage were not explored in depth, though they are well documented: in 2023, uptake among 15-year-old girls ranged from 67.7% in Brittany to 21.9% in Martinique [3]. One teacher noted that public and private schools follow the same guidelines, hinting at tensions between national recommendations and local implementation. Autonomy of regional education authorities may lead to unequal program delivery through variations in coordination, resources, or staff support. Future studies should include more geographically and socially diverse samples and examine how local contexts—urban vs. rural, governance structures—shape vaccine uptake and reinforce territorial inequalities.

The timing of the data collection is another element to consider. Conducted post-COVID-19 and during the rollout of France’s school-based HPV vaccination, interviews may have been influenced by this broader context. While some participants described strengthened trust in vaccination, others expressed fatigue or skepticism toward public health messaging. This makes it difficult to disentangle perceptions of the HPV vaccine from general pandemic experiences. Follow-up studies at greater distance from COVID-19 could help clarify this effect.

Finally, our sample did not include key vaccination stakeholders such as GPs, pediatricians, or midwives—despite their central role in influencing decisions. A longitudinal cohort including adolescents, parents, and a diverse range of healthcare professionals could capture how knowledge, attitudes, and decisions evolve over time, and assess the durability of effects from school-based campaigns, policy changes, and repeated professional recommendations (i.e., timing and completion).

## Conclusions

Beyond identifying barriers, our findings highlight actionable levers to strengthen HPV vaccination efforts. A clear message across groups was the need to enhance the school’s role—not just as a logistical site, but as a trusted platform for delivering clear, age-appropriate, and non-stigmatizing information. Building on the patterns described above should be structured, institutionally endorsed, and available to all teachers, with voluntary uptake, with clear communication tools and institutional backing to legitimize and normalize discussions. This could take the form of teacher-targeted toolkits co-developed with health professionals, including ready-to-use presentation materials, short training videos, and structured Q&A sheets [75]. Regular workshops or continuing education modules on HPV and adolescent vaccination could also be implemented through partnerships between regional health agencies and education authorities.

To conclude, increasing HPV vaccine uptake requires more than structural access—it demands institutional readiness, provider and family engagement, and youth empowerment. France’s school-based model offers a solid foundation, reinforced in 2024 by a second national campaign. This initiative enhanced interministerial coordination, improved early communication with families, and simplified consent and scheduling procedures [27], directly addressing concerns voiced by parents and school staff in our study. The campaign also expands communication strategies—using accessible, low-literacy materials and professional resources—and promotes the proactive involvement of local health actors (e.g., pharmacists, GPs, nurses, midwives; [27]). These developments signal a shift toward a more integrated, responsive prevention model. While long-term impact remains to be evaluated, research like the PREVHPV^9^ project, led by the French Institute for Public Health Research (IReSP), has demonstrated the willingness of French authorities to evaluate campaigns and inform future policy. Ultimately, international evidence and our findings converge in underscoring emotional literacy, trust in local providers, and tailored communication as critical levers for both effectiveness and equity.

## Supplementary Information

Supplementary Material 1.

## Data Availability

The data that support the findings of this study are available on request from the corresponding author. Due to the sensitive and confidential nature of participants’ responses, the data cannot be made publicly available.

## Abbreviations

HPV: Human papillomavirus
EU: European Union
HAS: Haute Autorité de Santé
GP: General practitioner
STI: Sexually transmitted infection
CPAM: Caisse Primaire d’Assurance Maladie
ARS: Agence Régionale de Santé
FLS: Français Langue Seconde
NVivo: Qualitative data analysis software (NVivo 15)
COM-B: Capability, Opportunity, Motivation—Behavior
CFIR: Consolidated Framework for Implementation Research

## Footnotes

1 Participants were recruited via a professional panel provider, which ensured that the sample met our predefined inclusion criteria and aimed to assemble a group that was as geographically and socio-demographically representative as possible. However, because the detailed breakdown of these characteristics was not made available to the research team, no further analyses could be conducted on this aspect.

2 This recruitment format involved 22 people, of whom 3 responded positively and agreed to the interview.

3 The participant’s reference to swimming pools reflects a common misconception regarding HPV transmission. HPV is not transmitted through pool water. However, several studies indicate that non-sexual routes of transmission are possible for certain cutaneous HPV types, including via skin-to-skin contact or contaminated surfaces (fomites). These mechanisms have been discussed in reviews examining the viability of HPV outside the host and its potential indirect transmission [77].

4 Primary Health Insurance Fund (Caisse primaire d’assurance maladie, CPAM).

5 This quotation reflects the participant’s spontaneous wording. The interviewer did not prompt, encourage, or frame the comment in any way.

6 Conization (also called a cone biopsy) is a surgical procedure in which a cone-shaped portion of cervical tissue is excised to diagnose and/or treat cervical intraepithelial neoplasia. When the lesion is fully removed, conization serves both to confirm histological grade and to prevent progression to cervical cancer [78, 79].

7 The use of summary leaflets, frequently mentioned by our participants, has also been strongly endorsed by general practitioners and pharmacists in other countries such as New Zealand [80].

8 A velfie (video selfie) is a short, self-recorded video where individuals share personal messages directly with an audience.

9 PREVHPV (Prévention des infections à papillomavirus humains) is a national French research project launched in 2022, coordinated by Université Paris-Saclay and Inserm, with support from IReSP and Santé publique France. It aims to evaluate the effectiveness of multi-level, population-based interventions to improve HPV vaccination coverage among adolescents. The project combines school-based actions, communication strategies tailored to adolescents and parents, and the involvement of healthcare professionals. PREVHPV includes both qualitative and quantitative assessments and is expected to provide evidence-based recommendations to guide future public health strategies on HPV prevention.
