# Supplementary material for: Perceptions of HPV vaccination: a qualitative study with adolescents, parents, school staff, and pharmacists
Source: BMC Med. 2026 Mar 26;24:291. doi: 10.1186/s12916-026-04821-z (PMC13141285; doi:10.1186/s12916-026-04821-z)
Supplement: Supplementary file 1 — Supplementary Material 1. [file 12916_2026_4821_MOESM1_ESM.docx]

Supplementary Material

## 1. Informed Consent Form for Parents (Child’s Participation)

Dear Sir/Madam,

You are invited to participate in the FRENCH-VACS research project, which aims to better understand the knowledge, opinions, and experiences of different stakeholders involved in HPV vaccination in middle schools. The goal is to improve the information and support provided to students and their families concerning this vaccination.

*Why this study?*
Vaccines play a crucial role in preventing serious diseases. This study seeks to collect your child’s viewpoint on vaccination in general, and on the HPV vaccine in particular, in order to identify factors influencing vaccination decisions.

*Your child’s participation*
We would like your child to participate in a 20–45 minute individual interview, conducted remotely. This interview will address the following topics:

- What your child knows or has heard about the HPV vaccine,
- Your child’s personal opinion, questions, or concerns about this vaccine,
- The elements that could influence your child’s decision, or that of their peers, concerning vaccination.

Your child’s participation is entirely voluntary, and their responses will be treated as strictly confidential and anonymous. There are no right or wrong answers, and they are encouraged to speak freely. You are also encouraged to discuss this participation with your child.

*Commitment of the interviewer*
The interviewer commits to conducting this research in accordance with ethical and professional provisions, protecting the physical, psychological, and social integrity of participants throughout the research, and ensuring confidentiality of the information collected. The interviewer also commits to providing participants with all necessary support to mitigate any negative effects that may result from participation in this research.

*Freedom of participants*
Consent to continue with the research may be withdrawn at any time, without giving a reason and without incurring any liability or consequence. Answers to questions are optional, and the absence of an answer has no consequence.

*Information for participants*Participants may obtain additional information regarding this study from the interviewer, within the limits of the research design.

*Confidentiality of information*Your child’s responses will be treated as strictly confidential and anonymous, and will be used only within the framework of this scientific research. Raw data will be securely stored for a period of 5 years, then destroyed. Under no circumstances will they allow the identification of your child. The results of the study may be published in scientific journals or presented at conferences, but your child’s anonymity will be preserved.

*Deontology and ethics*The interviewer commits to absolutely preserving confidentiality for all information concerning participants (Title I, Articles 1, 3, 5, and 6; and Title II, Articles 3, 9, and 20 of the Code of Ethics of Psychologists, France).

*Your consent*By authorizing your child to participate in this interview, you declare that you have read the information above and consent to their participation in our study. You are free to withdraw your consent at any time, without consequence for you or your child.

I, the undersigned __________________, freely and knowingly agree that my child participates in the study on human papillomavirus (HPV) vaccination.

Done at __________ on __________

Please indicate your choice below:

- ☐ I authorize my child to participate in the interview.
- ☐ I do not authorize my child to participate in the interview.

Date: __________
 Signature indicating participant’s consent: __________________

## 2. Informed Consent Form for Parents (Own Participation)

Dear Sir/Madam,

You are invited to participate in the FRENCH-VACS research project, which aims to better understand the knowledge, opinions, and concerns of different stakeholders involved in HPV vaccination in middle schools. The goal is to improve the information and support provided to students and their families concerning this vaccination.

*Why this study?*
Vaccines play a crucial role in preventing serious diseases. This study seeks to collect your viewpoint on vaccination in general, and on the HPV vaccine in particular, in order to identify the factors influencing vaccination decisions.

*Your participation*
We would like to interview you in the framework of a 20–45 minute individual interview, conducted remotely. This interview will address the following topics:

- Your general knowledge and attitudes toward vaccination,
- Your knowledge and reaction to the HPV vaccine,
- The factors influencing your decision regarding your child’s vaccination,
- The influence of recommendations from healthcare professionals and exchanges with other parents,
- Your perception of the school’s role in providing vaccination information,
- Your needs in terms of information and communication on vaccination.

Your participation is entirely voluntary, and your responses will be treated as strictly confidential and anonymous. There are no right or wrong answers, and you are encouraged to speak freely.

*Commitment of the interviewer*
The interviewer commits to conducting this research in accordance with ethical and professional provisions, protecting the physical, psychological, and social integrity of participants throughout the research, and ensuring confidentiality of the information collected. The interviewer also commits to providing participants with all necessary support to mitigate any negative effects that may result from participation in this research.

*Freedom of participants*
Consent to continue with the research may be withdrawn at any time, without giving a reason and without incurring any liability or consequence. Answers to questions are optional, and the absence of an answer has no consequence.

*Information for participants*
Participants may obtain additional information regarding this study from the interviewer, within the limits of the research design.

*Confidentiality of information*
Your responses will be treated as strictly confidential and anonymous, and will be used only within the framework of this scientific research. Raw data will be securely stored for a period of 5 years, then destroyed. Under no circumstances will they allow your identification. The results of the study may be published in scientific journals or presented at conferences, but your anonymity will be preserved.

*Deontology and ethics*
The interviewer commits to absolutely preserving confidentiality for all information concerning participants (Title I, Articles 1, 3, 5, and 6; and Title II, Articles 3, 9, and 20 of the Code of Ethics of Psychologists, France).

*Your consent*
By agreeing to participate in this interview, you declare that you have read the information above and consent to participate in our study. You are free to withdraw your consent at any time, without consequence for you.

I, the undersigned __________________, freely and knowingly agree to participate in the study on human papillomavirus (HPV) vaccination.

Done at __________ on __________

Please indicate your choice below:

- ☐ I agree to participate in the interview.
- ☐ I do not wish to participate in the interview.

Date: __________
 Signature indicating participant’s consent: __________________

## 3. Informed Consent Form for School Staff

Dear Sir/Madam,

You are invited to participate in the FRENCH-VACS research project, which aims to better understand the knowledge, opinions, and experiences of different stakeholders involved in HPV vaccination in middle schools. The goal is to improve the information and the implementation of vaccination campaigns in school settings.

*Why this study?*
Vaccines play a crucial role in preventing serious diseases, and school is an important place for information and awareness-raising. This study seeks to collect your viewpoint as a member of school staff on vaccination in general, and on the HPV vaccine in particular, in order to identify factors influencing vaccination uptake and the role of the school in this domain.

*Your participation*
We would like to interview you in the framework of a 20–45 minute individual interview, conducted remotely. This interview will address the following topics:

- Your perception of the school’s role in informing and promoting vaccination,
- Your observations on students’ and parents’ attitudes and reactions concerning HPV vaccination,
- Your experience in communicating about vaccination with students and families,
- Your needs in terms of resources, training, or materials to strengthen information and trust around this vaccine.

Your participation is entirely voluntary, and your responses will be treated as strictly confidential and anonymous. There are no right or wrong answers, and you are encouraged to speak freely.

*Commitment of the interviewer*
The interviewer commits to conducting this research in accordance with ethical and professional provisions, protecting the physical, psychological, and social integrity of participants throughout the research, and ensuring confidentiality of the information collected. The interviewer also commits to providing participants with all necessary support to mitigate any negative effects that may result from participation in this research.

*Freedom of participants*
Consent to continue with the research may be withdrawn at any time, without giving a reason and without incurring any liability or consequence. Answers to questions are optional, and the absence of an answer has no consequence.

*Information for participants*
Participants may obtain additional information regarding this study from the interviewer, within the limits of the research design.

*Confidentiality of information*
Your responses will be treated as strictly confidential and anonymous, and will be used only within the framework of this scientific research. Raw data will be securely stored for a period of 5 years, then destroyed. Under no circumstances will they allow your identification. The results of the study may be published in scientific journals or presented at conferences, but your anonymity will be preserved.

*Deontology and ethics*
The interviewer commits to absolutely preserving confidentiality for all information concerning participants (Title I, Articles 1, 3, 5, and 6; and Title II, Articles 3, 9, and 20 of the Code of Ethics of Psychologists, France).

*Your consent*
By agreeing to participate in this interview, you declare that you have read the information above and consent to participate in our study. You are free to withdraw your consent at any time, without consequence for you.

I, the undersigned __________________, freely and knowingly agree to participate in the study on human papillomavirus (HPV) vaccination.

Done at __________ on __________

Please indicate your choice below:

- ☐ I agree to participate in the interview.
- ☐ I do not wish to participate in the interview.

Date: __________
 Signature indicating participant’s consent: __________________

## 4. Informed Consent Form for Pharmacists

Dear Sir/Madam,

You are invited to participate in the FRENCH-VACS research project, which aims to better understand the role of pharmacists in HPV vaccination. The objective is to identify the levers and obstacles to pharmacists’ involvement in promoting HPV vaccination and to optimize their contribution to this public health effort.

*Why this study?*
Pharmacists play an important, close-to-the-public role and are increasingly involved in vaccination. This study seeks to collect your viewpoint and experience with HPV vaccination, in order to identify ways to better support you in this mission.

*Your participation*
We would like to interview you in the framework of a 20–45 minute individual interview, conducted remotely. This interview will address the following topics:

- Your perception of your role in HPV vaccination and its evolution,
- Your interactions with patients regarding HPV vaccination, including the questions and concerns they express,
- Your evaluation of your training and level of preparedness to answer patients’ questions and promote vaccination,
- The obstacles and facilitators you identify in your daily practice concerning HPV vaccination.

Your participation is entirely voluntary, and your responses will be treated as strictly confidential and anonymous. There are no right or wrong answers, and you are encouraged to speak freely.

*Commitment of the interviewer*
The interviewer commits to conducting this research in accordance with ethical and professional provisions, protecting the physical, psychological, and social integrity of participants throughout the research, and ensuring confidentiality of the information collected. The interviewer also commits to providing participants with all necessary support to mitigate any negative effects that may result from participation in this research.

*Freedom of participants*
Consent to continue with the research may be withdrawn at any time, without giving a reason and without incurring any liability or consequence. Answers to questions are optional, and the absence of an answer has no consequence.

*Information for participants*Participants may obtain additional information regarding this study from the interviewer, within the limits of the research design.

*Confidentiality of information*
Your responses will be treated as strictly confidential and anonymous, and will be used only within the framework of this scientific research. Raw data will be securely stored for a period of 5 years, then destroyed. Under no circumstances will they allow your identification. The results of the study may be published in scientific journals or presented at conferences, but your anonymity will be preserved.

*Deontology and ethics*
The interviewer commits to absolutely preserving confidentiality for all information concerning participants (Title I, Articles 1, 3, 5, and 6; and Title II, Articles 3, 9, and 20 of the Code of Ethics of Psychologists, France).

*Your consent*
By agreeing to participate in this interview, you declare that you have read the information above and consent to participate in our study. You are free to withdraw your consent at any time, without consequence for you.

I, the undersigned __________________, freely and knowingly agree to participate in the study on human papillomavirus (HPV) vaccination.

Done at __________ on __________

Please indicate your choice below:

- ☐ I agree to participate in the interview.
- ☐ I do not wish to participate in the interview.

Date: __________
 Signature indicating participant’s consent: __________________

## 5. Students Interview Guide

1. **Understanding of vaccination** • Do you know what a vaccine is? Can you tell me what it does in your body?
    • Have you ever been vaccinated at school or at the doctor’s office? Do you remember what it was like?
2. **Knowledge and opinions about the HPV vaccine** • Have you heard about the HPV vaccine? Where? From whom?
    • Has this vaccine ever been offered to you? If yes, how did you feel? (uncertain, scared, confident…)
3. **Sources of information and influence** • When you hear about vaccines, who talks to you about them most often?
    • Does what adults say (parents, teachers, doctors…) matter to you? And your friends?
4. **Reasons to accept or refuse a vaccine** • What makes you want to get vaccinated?
    • What might make you hesitate or say no?
5. **Role of the school and professionals** • Do you talk about vaccination in class or at school?
    • Would you like teachers or the school nurse to explain vaccines to you more? Why?

## 6. Parents Interview Guide

1. **General knowledge and attitudes** • What is your general perception of vaccines today?
    • Had you already heard about the HPV vaccine before this project? What was your reaction?
2. **Decision-making factors** • What led you to (not) have your child vaccinated against HPV?
    • What kinds of doubts or hesitations did you experience?
3. **Influence of the environment** • To what extent do recommendations from your doctor or pharmacist influence your choice?
    • Do you talk about this with other parents? What comes out of those discussions?
4. **Role of the school** • Do you think the school plays an important role in providing information on vaccination?
    • What do you think about vaccination campaigns organized directly in middle schools?
5. **Needs and expectations** • What information or types of communication would have helped you in your decision?
    • Do you have suggestions for better informing families?

## 7. School Staff Interview Guide

1. **Representation of vaccination** • What is your perception of the human papillomavirus (HPV) vaccine?
    • Have you been informed or trained about this vaccine as part of your work?
2. **Role of the school** • Do you think the school has a role to play in providing information or promoting vaccination?
    • Have you ever been approached by students or parents on this subject?
3. **Observations and interactions** • What attitudes do you notice among students or families?
    • What kinds of statements (positive or negative) do you hear?
4. **Needs and recommendations** • Would you like to have more resources, training, or materials?
    • In your opinion, what would be the best ways to strengthen information and trust around this vaccine?

## 7. Pharmacists Interview Guide

1. **Professional role and evolution** • How do you perceive your role in vaccination, particularly regarding HPV?
    • Have you noticed a change in demand or client attitudes since you started vaccinating?
2. **Interactions with patients** • What types of questions or concerns do your clients raise about this vaccine?
    • How do you respond to hesitancy or doubts?
3. **Training and support** • Do you feel sufficiently trained to answer patients’ questions?
    • What would you need in order to better support the public in this role?
4. **Obstacles and facilitators** • In your opinion, what are the main barriers to vaccination in your daily practice?
    • What works well to reassure or convince hesitant individuals?
